# Disease-associated XMRV sequences are consistent with laboratory contamination

**DOI:** 10.1186/1742-4690-7-111

**Published:** 2010-12-20

**Authors:** Stéphane Hué, Eleanor R Gray, Astrid Gall, Aris Katzourakis, Choon Ping Tan, Charlotte J Houldcroft, Stuart McLaren, Deenan Pillay, Andrew Futreal, Jeremy A Garson, Oliver G Pybus, Paul Kellam, Greg J Towers

**Affiliations:** 1 MRC Centre for Medical Molecular Virology, Division of Infection and Immunity, University College London, 46 Cleveland St, London W1T 4JF, UK; 2 Wellcome Trust Sanger Institute, Hinxton, Cambridge, CB10 1SA, UK; 3 Department of Zoology, University of Oxford, South Parks Road, Oxford, OX1 3PS, UK

## Abstract

**Background:**

Xenotropic murine leukaemia viruses (MLV-X) are endogenous gammaretroviruses that infect cells from many species, including humans. Xenotropic murine leukaemia virus-related virus (XMRV) is a retrovirus that has been the subject of intense debate since its detection in samples from humans with prostate cancer (PC) and chronic fatigue syndrome (CFS). Controversy has arisen from the failure of some studies to detect XMRV in PC or CFS patients and from inconsistent detection of XMRV in healthy controls.

**Results:**

Here we demonstrate that Taqman PCR primers previously described as XMRV-specific can amplify common murine endogenous viral sequences from mouse suggesting that mouse DNA can contaminate patient samples and confound specific XMRV detection. To consider the provenance of XMRV we sequenced XMRV from the cell line 22Rv1, which is infected with an MLV-X that is indistinguishable from patient derived XMRV. Bayesian phylogenies clearly show that XMRV sequences reportedly derived from unlinked patients form a monophyletic clade with interspersed 22Rv1 clones (posterior probability >0.99). The cell line-derived sequences are ancestral to the patient-derived sequences (posterior probability >0.99). Furthermore, *pol *sequences apparently amplified from PC patient material (VP29 and VP184) are recombinants of XMRV and Moloney MLV (MoMLV) a virus with an envelope that lacks tropism for human cells. Considering the diversity of XMRV we show that the mean pairwise genetic distance among *env *and *pol *22Rv1-derived sequences exceeds that of patient-associated sequences (Wilcoxon rank sum test: p = 0.005 and p < 0.001 for *pol *and *env*, respectively). Thus XMRV sequences acquire diversity in a cell line but not in patient samples. These observations are difficult to reconcile with the hypothesis that published XMRV sequences are related by a process of infectious transmission.

**Conclusions:**

We provide several independent lines of evidence that XMRV detected by sensitive PCR methods in patient samples is the likely result of PCR contamination with mouse DNA and that the described clones of XMRV arose from the tumour cell line 22Rv1, which was probably infected with XMRV during xenografting in mice. We propose that XMRV might not be a genuine human pathogen.

## Background

XMRV (Xenotropic murine leukaemia virus-related virus) is a xenotropic murine leukaemia virus (MLV-X) that has been detected in samples from prostate cancer (PC) and chronic fatigue syndrome (CFS) patients[1-6]. This has led to the suggestion that infection with this virus might cause these conditions. MLV-Xs are endogenous gamma retroviruses found in the genomes of mice. They are so named because *in vitro *they infect cells from a variety of species but were originally found not to infect the inbred strains of mice from which they were derived, due to mutations in the host xenotropic receptor. More recently, murine xenotropic receptor variants have been described which support MLV-X infection revealing a complex evolutionary relationship between MLV-X envelope sequences and their receptors in rodents [7-9]. XMRV has also been detected in 1-6% of healthy human controls in some studies, suggesting that infection may be common in the healthy human population [2,3,5]. The association between XMRV and human disease is controversial, with some studies detecting XMRV in up to 67% of patients whilst others have failed to detect XMRV infection [10-18]. Importantly, examination of infected prostate tumours reveals that not all the tumour cells are infected with XMRV suggesting that XMRV insertion is not required for tumourogenesis [1]. XMRV sequences detected in patients are remarkably similar to each other often differing by only a few nucleotides between unlinked patients [2]. This lack of sequence variation appears inconsistent with a retrovirus infecting geographically separated, unconnected individuals. Here, we have examined the specificity of XMRV PCR, XMRV sequence variation and the phylogenetic relationship between XMRV detected in humans and as contaminants in cell culture. We conclude that XMRV in patient samples is likely to be derived from PCR contamination from either mouse DNA or cell lines infected with XMRV, and that XMRV is unlikely to be a human pathogen.

## Results and Discussion

### Primers reported to be XMRV specific can detect mouse DNA

**Figure 1:**
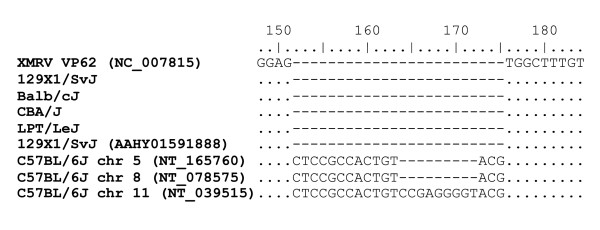
**Alignment of XMRV *gag*-leader sequence with *gag*-leader sequences from endogenous MLVs in mice**. XMRV-like sequences from four inbred mice containing the 24 nt deletion signature were identified by deep sequencing and one sequence identified by BLAST. The most similar sequences from the C57BL/6 genome are shown for comparison and all sequences are compared to XMRV VP62. Numbering refers to the length of the PCR product derived using primers EG87 and EG89 (Additional File 1; Table S1).

To better understand the provenance of XMRV [1,2] we screened nine inbred and three wild-derived inbred mouse strains with Taqman PCR primers previously used to specifically detect XMRV. We selected the mouse lines to be widely spread across the inbred genealogy [19] and to be available as DNA from the JAX database, Jackson Laboratories Bar Harbor, Maine. We first used primers targeting a 24 nt deletion in the *gag*-leader region reported to be XMRV-specific [1,4]. Significantly, all 12 mouse strains were PCR positive (Table 1). We also detected this reportedly-specific deletion in the *gag*-leader of endogenous proviruses in 4 mouse strains (129X1/SvJ, Balb/cJ, CBA/J and LPT/LeJ) by 454 deep sequencing (Roche) the PCR product amplified with primers flanking the deletion (Figure 1 Table 1 and Additional File 1; Table S1). The deletion was at a low frequency, consistent with it being present in just one (or a few) of many endogenous proviral copies compared to other murine leukaemia viruses (MLVs) present in higher copy numbers. Since some Taqman PCR-positive mice were negative for this 24 nt *gag*-leader deletion by deep sequencing, we conclude that either these Taqman primers are not specific for the deletion, or that endogenous murine leukaemia virus (MLV) sequences with the deletion were not always PCR-amplified in this deep sequencing experiment, possibly due to primer mismatch. We certainly cannot compare deep sequencing with Taqman PCR in terms of sensitivity, but both of these techniques suggest that the *gag*-leader deletion can be found in the genome of some inbred mouse strains. We found further evidence for this XMRV signature sequence in GenBank: a 1124 nt sequence encoding the "XMRV-specific" *gag*-leader 24 nt deletion is present in the genome of 129X1/SvJ strain mice (AAHY01591888 Figure 1). We also tested the specificity of XMRV *integrase *Taqman primers previously used to screen for XMRV [5]. Amplification of mouse genomic DNA showed high copy (2 strains), low copy (6 strains) and undetectable (4 strains) levels of amplifyable MLV provirus using these primers (Table 1). These data indicate that primer sets previously described as XMRV-specific can readily amplify MLV sequences from a variety of mice when used under the PCR conditions described [4,5,14], and that some targets exist at high copy number in genomes of mice.

**Table 1:** Screening of genomic DNA from 9 inbred and 3 wild-derived inbred mice using Taqman PCR

Mouse strain				Taqman PCR result (Ct FAM)		
			
Name	Jackson ID	454 deep sequencing		XMRV - *gag*-leader	XMRV - *int*	MLV-X - *gag*
		**Read depth**	**(%)**	**Ct**	**Ct**	**Ct**

***Inbred***						

129X1/SvJ	000691	64706	0.73	28	> 41	21

Balb/cJ	000651	32512	0.62	31	> 41	23

C57BL/6J	000664	39548	0	27	> 41	20

C57BR/cdJ	000667	29280	0	27	21	21

CBA/J	000656	20548	0.77	27	39	21

DBA/1J	000670	63559	0	28	35	20

I/LnJ	000674	8012	0	28	38	20

LPT/LeJ	000220	10303	0.64	28	> 41	22

NZW/LacJ	001058	11644	0	29	21	20

***Wild-derived inbred***						

PWK/PhJ	003715	9457	0	29	38	19

WMP/PasDnJ	001746	19294	0	28	36	22

WSB/EiJ	001145	14062	0	38	38	22

Mice names and Jackson lab identification numbers (ID) are shown as are the proportion of positive 454-sequencing reads that contain the XMRV 24 nt deletion signature sequence compared to the total number of reads obtained from each amplification reaction. Cycle threshold (Ct) values for Taqman PCR performed with primer sets targeting XMRV *gag*-leader, *integrase *and MLV-X-*gag *are also shown. Template amounts were 1 ng, 200 ng and 2 ng respectively. Lower amounts of genomic DNA were used in *gag *and *gag*-leader PCRs due to the high number of amplicons detected. We define positive PCR as those with a Ct of less than 41. This cut off was chosen on the basis that PCR quantitation of plasmid concentrations of 5 molecules per PCR give Ct values of 40. PCR detection of concentrations below 5 molecules became stochastic and are thus below the limit of reliable detection (data not shown). Primers are shown in Table S1.

### Human cell lines are commonly contaminated with xenotropic MLVs

Human cell lines have been found contaminated with gammaretroviruses including xenotropic murine leukaemia viruses (MLV-X) [20,21]. They are likely to have been transmitted to human cells during cell passage as grafts in mice, or when human cells are cultured together with mouse cells. In order to explore the frequency and genetic diversity of XMRV-like sequences in cell culture, we screened 411 cell lines from the COSMIC collection (Additional File 2; Table S2) [22]. We chose this collection as a source of well characterized human tumour cell lines of different tumour types. We used Taqman primers for the XMRV *gag*-leader deletion[4], the XMRV *integrase *[5], and also used primers designed to amplify diverse MLV-X *gag *sequences[14] (Additional File 1; Table S1). Nine human cell lines (2.2%) were positive using MLV-X-*gag *primers (Table 2). Five of these nine lines were also positive using XMRV *gag*-leader primers[4]; but none were positive using XMRV-*integrase *primers [5] (Table 2). Direct sequencing of *gag*, *pol *and *env *PCR products amplified from these cell lines revealed a single sequence in most cases (Table 2). Phylogenetic analysis of these sequences confirmed that the contaminating viruses are closely related to MLV-X previously found infecting cultured human cell lines[20] (Additional File 3; Figure S1). Importantly, MLV-X viruses in human cell lines, including XMRV, are contained within the genetic diversity of known murine viruses and do not represent an outgroup or a specific clade that is more common in human tumour cell lines. Thus human cell lines commonly carry retroviruses that can be amplified with primers erroneously described as specific to XMRV [4,5].

**Table 2:** Screening human cell line genomic DNA using Taqman PCR

Cancer cell line			Taqman PCR Result (Ct FAM)			Sequences obtained		
**Name**	**COSMIC ID**	**Cancer type**	**XMRV -** ***gag*-leader**	**XMRV *-int***	**MLV-X -*gag***	***gag***	***pol***	***env***

A2780	906804	Ovary adenocarcinoma	No Ct	No Ct	27	S	S	S

BHY	753535	Squamous cell carcinoma	No Ct	No Ct	15	S	S	S

CoCM-1	910783	Colon adenocarcinoma	16	No Ct	16	S	S	M

Daudi	906831	Lymphoid neoplasm/Burkitt lymphoma	21	No Ct	22	M	M	S

EKVX	905970	Lung adenocarcinoma	No Ct	No Ct	18	M	S	S

IMR-5	907170	Neuroblastoma	No Ct	No Ct	20	S	S	S

MUTZ-1	908155	Haematopoietic neoplasm/Myeloid leukaemia	40	No Ct	14	S	S	S

S-117	910946	Thyroid sarcoma	19	No Ct	18	M	S	S

TYK-nu	909774	Ovary carcinoma	27	No Ct	18	M	S	S

Only MLV-X-*gag *positive cell lines are included. A total of 411 DNAs from human tumour cell lines from the COSMIC (Catalogue Of Somatic Mutations In Cancer repository) http://www.sanger.ac.uk/genetics/CGP/cosmic representing a variety of cancer types were screened. Common names, (Cosmic ID) and tumour type are shown. Cycle threshold (Ct) values for the three Taqman PCR reactions are shown. (S) denotes a single sequence (M) denotes multiple sequences obtained after direct sequencing of *gag, pol *and *env *PCR products. We define positive PCR as those with a Ct of less than 41. Primers are shown in Table S1.

### Phylogenetic analysis of XMRV sequences

Analysis of the genetic diversity and phylogenetic relationships among retroviral sequences, both endogenous [23] and exogenous [24] can reveal information about their replication and evolutionary history. We therefore performed extensive evolutionary analysis of published XMRV and related sequences in order to better understand their origin and proliferation. The widely studied prostate cancer line 22Rv1 is reported to produce high levels of a virus closely related to XMRV [25,26]. We therefore cloned and sequenced *gag *(n = 16), *pol *(n = 18) and *env *(n = 10) PCR products amplified from genomic DNA purified from this cell line. Of these, 13/16 *gag *sequences, 15/18 *pol *sequences and 8/10 *env *sequences were unique. These numbers are consistent with the previously estimated XMRV copy number in the 22Rv1 cell line of 10-20 copies [25]. We analysed these unique cell line sequences together with (i) previously-described full-length endogenous MLV genomes[27] (n = 46), (ii) a previously described XMRV clone from the 22Rv1 cell line (n = 1 [26] GenBank: FN692043) (iii) full-length XMRV sequences reportedly amplified from PC (n = 6)[1], or CFS patient samples (n = 2)[2], (iv) previously reported XMRV *pol *sequences derived from PC patient material (n = 6)[1], (v) additional C57BL/6 endogenous full-length MLV sequences identified using BLAT (n = 28), and (vi) various other MLV complete genomes (n = 5).

Quite unexpectedly, visual inspection of the 2552 nt XMRV *pol *sequences [1] revealed that sequences VP29 and VP184, which were apparently amplified from PC patient material, are recombinants of the 22Rv1 cell line virus and Moloney MLV (MoMLV). A nucleotide BLAST search revealed that the Moloney MLV derived fragment from VP29 (1182 nt) is 100% identical to MoMLV (GenBank AF033811), 11 nucleotides different to the closest known mouse endogenous MLV (GenBank AC153360) and 22 nucleotides different to the XMRV clone derived from the 22Rv1 cell line (FN692043). The recombinant nature of VP29 and VP184 was confirmed by phylogenetic incongruence analysis (Additional File 4; Figure S2). The fact that MoMLV envelope does not have tropism for human cells, that these PCR products were derived from human material, and that the 1182 nt XMRV fragment is identical to common MLV-based plasmids, strongly suggests PCR contamination as the source of the recombinant.

**Figure 2:**
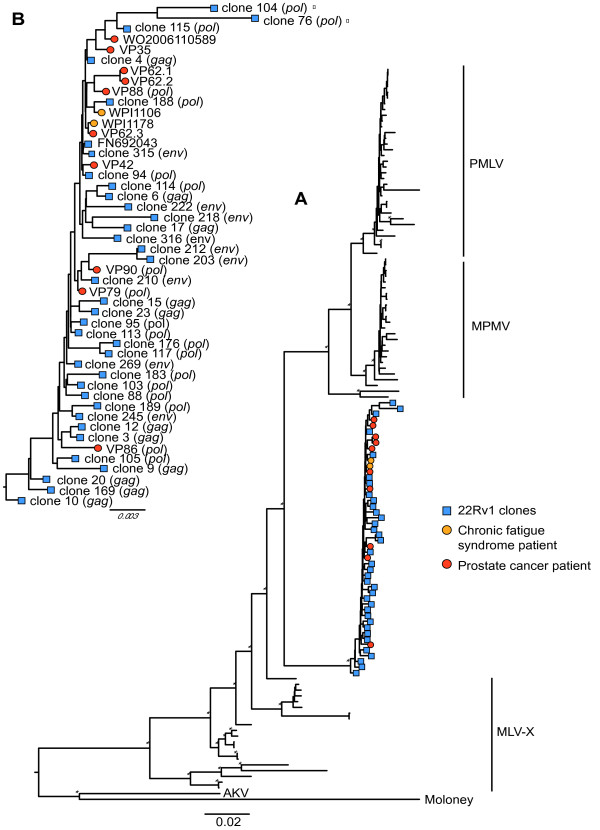
**Phylogeny of 22Rv1 and patient derived XMRV sequences and other murine leukaemia viruses**. Bayesian maximum clade credibility phylogeny of 22Rv1 cell line derived XMRV clones, patient derived XMRV sequences and other murine leukaemia viruses **(A)**. The monophyletic cluster formed by 22Rv1 cell line derived XMRV clones and patient derived XMRVs is magnified in **(B)**. Xenotropic MLV (MLV-X), polytropic MLV (PMLV), and modified polytropic MLV (MPMLV) were added as controls. Sequences derived from prostate cancer patients (VP and WO) and chronic fatigue syndrome patients (WPI) are indicated by red and yellow circles respectively. Gene sequences derived from 22Rv1 clones are indicated by blue squares. When full-length genomes were not available, the loci of the sequence used in the phylogenetic reconstruction are shown in brackets. APOBEC-3G/F hypermutated clones are labelled with a closed circle. The tree is rooted against AKV and Moloney MLVs. Bayesian posterior probabilities of 1.00 are indicated on the corresponding branches by a star. The scale bar represents the number of nucleotide substitutions per site.

Next, we investigated the evolutionary relationships among the aforementioned sequences (excluding identical 22Rv1 clone sequences and the recombinants) using Bayesian phylogenetic methods. The resulting phylogeny (Figure 2) clearly shows that XMRV sequences reportedly derived from unlinked patients are interspersed among sequences derived from the 22Rv1 cell line within a single strongly supported monophyletic cluster (posterior probability >0.99; Figure 2). These results were consistent when phylogenies were reconstructed on the basis of the *gag*, *pol *and *env *gene independently (see Additional File 5; Figure S3). In addition to the interspersion of cell line and patient derived sequences, cell line-derived sequences are basal to the patient-derived sequences (Figure 2). However, many of the XMRV and XMRV related sequences are so closely related to each other that the precise branching order within the XMRV cluster could not be elucidated with robust support. As a result no one particular clone could be identified as the ancestor of the cluster with high statistical support in either the full length (Figure 2) or gene specific (Additional File 5; Figure S3) trees. To examine this further, we inspected the 3000 most probable Bayesian trees obtained from the full-length alignment (Figure 2) and found that a cell line derived sequence was basal to the XMRV cluster in every case (data not shown). Thus, the estimated posterior probability that the ancestor of the cluster was not a cell line derived sequence was <0.001. Together these observations support the notion that the 22Rv1 cell line XMRV sequences are ancestral to the patient-associated XMRV sequences in this analysis. We have used the tree constructed from full-length and non-overlapping fragments rather than the gene specific trees (Additional File 5; Figure S3) in order to include all the available variation within the XMRV sequences in the analysis. Non-overlapping sequences will not induce a bias in the Bayesian phylogenetic reconstruction as long as they are individually compared to full-length genomes.

### 22Rv1 associated XMRV is more diverse than patient derived sequences

**Figure 3:**
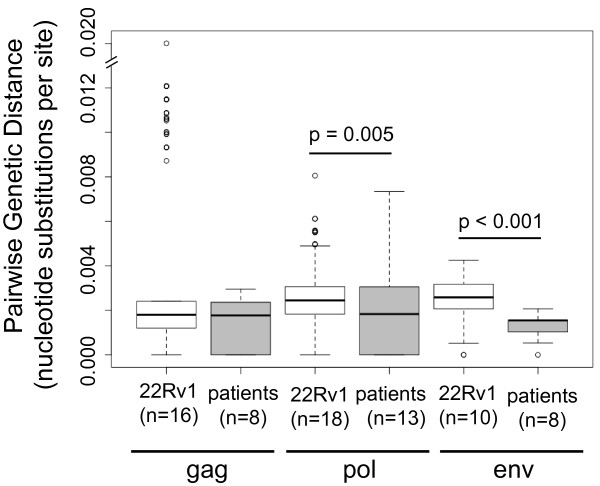
**Enumeration of nucleotide substitutions per site between the cell-line and patient-derived XMRV sequences **. The pairwise genetic distance among *pol *and *env *sequences derived from 22Rv1 cells (white boxes) is significantly higher than among patient-associated sequences (grey boxes) (Wilcoxon rank sum test: p = 0.005 and p < 0.001 respectively). There is no significant difference in variation in the *gag *region. The top end of the *y*-axis was truncated to accommodate outliers in the *gag *22Rv1 category. Outliers are due to APOBEC hypermutation.

The observed genetic diversities of cell-line and patient-derived sequences are also difficult to reconcile with the hypothesis that published XMRV sequences are related by a process of infectious transmission. The mean pairwise genetic distance among *pol *and *env *gene sequences derived from 22Rv1 cells exceeds that among patient-associated sequences (Wilcoxon rank sum test: p = 0.005 and p < 0.001 for *pol *and *env*, respectively; Figure 3 and Additional File 6; Table S3). For the *gag *region, the mean pairwise genetic diversities of patient-derived and cell-line sequences are not significantly different (Figure 3 and Additional File 6; Table S3). In order to test for the potential confounding factor of PCR and sequencing errors in the 22Rv1 clones diversity, genetic distances were re-calculated assuming that 1% of the diversity seen in the clones was artefactual. Even under such an extreme scenario, and assuming no sequencing error in the patient-derived sequences, the mean pairwise genetic diversities of patient-derived and cell-line sequences are not significantly different in the *gag *and *pol *loci, while the genetic distance among *env *gene sequences derived from 22Rv1 cells still exceeds that among patient-associated sequences (Wilcoxon rank sum test: p < 0.001; data not shown). Even under the most conservative hypothesis that XMRV undergoes almost no evolutionary change upon transmission, we would expect sequences sampled from geographically-disparate individuals, with no known epidemiological linkage, to exhibit more diversity than sequences derived from a single infected cell line. We cannot reject the possibility that cell line-associated XMRV diversity is higher because it has undergone more replication than XMRV in epidemiologically-unlinked individuals in different disease cohorts or that the patients were infected by a clonal virus from an unidentified source. However, to our knowledge, there are no examples of reported accelerated viral evolution in culture as compared to in natural hosts and therefore in the context of our other results, this seems unlikely.

Another notable characteristic of the XMRV clade is its asymmetry (B_1 _asymmetry statistic = 24.47, p < 0.002). This is an expected property of families of endogenous mobile elements [23]. Phylogenetic asymmetry implies that whenever replication occurs, one daughter sequence tends to be inactive whilst the other continues to proliferate. This phenomenon arises naturally when one (or a few) active endogenous viruses in a genome generate inactive copies by re-infection [23], but is difficult to explain under a hypothesis of host-to-host transmission. Extreme cases of strong selection among genetically diverse variants can cause asymmetry [28], although in this case, the lack of XMRV genetic diversity is incompatible with this possibility.

Whilst our observations cannot conclusively prove that XMRV is not a human pathogen they appear consistent with the hypothesis that XMRV is not an exogenous virus transmitting among individuals. Instead, multiple lines of evidence suggest that the full length clones of XMRV originated from the 22Rv1 cell line. PCR contamination could arise directly from 22Rv1 cells or from cells inadvertently infected with the 22Rv1 derived virus. We speculate that the 22Rv1 cells became infected with XMRV during their passage through athymic mice [29]. Data in Figure 1 demonstrate that mouse DNA could also contaminate patient samples as a variety of mice encode sequences, with endogenous MLV proviruses, that are detected with PCR protocols that are designed to detect XMRV. It is quite possible therefore that previously published findings are explained by contaminated PCR where the patient samples were contaminated by mouse DNA or DNA from cells infected with MLV-X including that from 22Rv1 cells. A recent study amplified polytropic MLV sequences rather than XMRV from chronic fatigue patient samples [30] and healthy donors. Unfortunately the MLV sequences described there were too short to carry out a thorough phylogenetic analysis, and we have therefore not included them here. It is difficult to retrospectively establish whether prior studies have contaminated patient samples. Importantly, assay contamination cannot be assessed by detection of murine DNA alone since MLVs contaminate a significant proportion of non-murine cell lines common in laboratories [1,30]. PCR contamination has previously been found to underlie erroneous association between retroviruses and human disease underlining the difficulties associated with detecting pathogens by PCR [31,32].

## Conclusions

We conclude that future screens for MLV-related sequences use more rigorous PCR containment procedures, such as those used to reliably recover ancient DNA [33], or manage contamination by controlling for its inevitable frequency, for example by screening equal numbers of controls prepared and stored identically, together with test samples [34]. Positive samples must be sequenced and those that are identical to known endogenous murine sequences, or plasmids present in the host laboratory, should be treated with caution. Whilst true association of XMRV with human disease would be of great medical importance, it is imperative that such an association is rigorously established before it impacts on diagnosis and patient care. We suggest that XMRV as a human virus does not conform to this criterion.

## Methods

### Taqman PCR

PCR of mouse genomic DNA was performed using primers/probes as previously described (Additional File 1; Table S1) [4,5,14]. PCR conditions including buffers (a single batch of 2× Taqman PCR master mix (Applied Biosystems)) and thermocycler conditions were also essentially as described [4,5,14]. All human tumour cell line Taqman PCRs were run in a duplex assay using the Taqman^® ^RNase P Control Reagents (VIC) (Applied Biosystems) as an internal control. Cycling conditions were 95°C for 15 secs and annealing/extension at 60°C for 1 minute after an initial denaturation of 10 min. Thresholds were routinely set at default values. Primers are shown in Additional File 1; Table S1.

### 454 Sequencing of inbred and wild-derived inbred mouse samples

Mouse DNA samples were obtained from the Jackson Labs (Bar Harbor, Maine) except for Balb/c which was obtained from Sigma (D4416). 100-200 ng DNA from each mouse was amplified using Platinum *Pfx *(Invitrogen) proofreading polymerase and primers EG87 and EG89 (Additional File 1; Table S1). 500 ng of amplified DNAs were sequenced using the Genome Sequencer FLX Instrument and GS FLX Titanium series reagents (Roche/454 Life Sciences) according to the manufacturer's instructions. SFF files were processed using the sfffile and sffinfo commands of the SFF tools, split based on the MIDs, and FASTA files were created for each sample. Reads containing XMRV-specific 24-nt deletion, were identified using a customised python script and their frequency was calculated.

### PCR, direct sequencing and sequence analysis

Partial *gag, pol *and *env *sequences were amplified from genomic DNA from human tumour cell lines using primers labelled TC in Additional File 1; Table S1. PCR products were purified and subjected to direct sequencing, which obviated PCR error, using an Applied Biosystems 3730×l DNA analyzer. *Gag, pol *and *env *sequences from the 22Rv1 cell line were amplified with a single stock of Platinum *Pfx *(Invitrogen) proofreading polymerase and primers labelled 22Rv1 in Additional File 1; Table S1. PCR product was gel purified and ligated into pZero Blunt and transformed (Invitrogen). Positive plasmid clones derived from individual colonies were then sequenced.

### Phylogenetic analysis

22Rv1 cell line derived *gag *(1605 nt; n = 11), *pol *(1635 nt; n = 15) and *env *(1935; n = 8) unique sequences were manually aligned with 6 full-length XMRV sequences apparently amplified from PC samples (GenBank DQ241301, DQ241302, DQ399707, EF185282, FB579966, NC_007815), 2 full-length XMRV sequences from CFS patient samples (GenBank GQ497343, GQ497344), previously described *pol *sequences derived from PC patient material (VP29, VP79, VP86, VP88, VP90, VP184; n = 6)[1], one previously reported sequence derived from the 22Rv1 tumour cell line (GenBank FN692043) [26], 46 nonecotropic endogenous MLV sequences[27], 28 endogenised MLV full-length sequences identified by BLAT search [35] of the mouse genome using the VP62 XMRV sequence (GenBank DQ399707) as a query, the DG-75 MLV complete genome sequence (GenBank AF221065), one murine type C retrovirus complete genome (GenBank X94150) and one murine AIDS virus provirus complete sequence (GenBank S80082). The complete genome sequences of AKV and Moloney MLV (GenBank J01998 and AF033811) were added as outgroups. Bayesian phylogenies were reconstructed with the software MrBayes version 3.1.2 [36], under the General Time Reversible model of nucleotide substitution, with proportion of invariable sites and gamma-distributed rate heterogeneity (GTR+I+G). GTR+I+G parameters were estimated with the program PAUP* version 4b10[37] using full-length genomes only, and were fixed prior to the phylogenetic reconstruction. The Markov chain Monte Carlo (MCMC) search was set to 3,000,000 iterations, with trees sampled every 1000th generation. Maximum clade credibility trees were selected from the posterior distribution with the program TreeAnnotator version 1.5.2 http://beast.bio.ed.ac.uk/, after discarding a 20% burn in. Trees were edited with the program FigTree version 1.1.2 http://tree.bio.ed.ac.uk/software/figtree/. Phylogenetic reconstructions were also conducted in a gene-specific manner for the *gag*, *pol *and *env *loci following the aformentioned methodology. The posterior probability that the ancestor of the XMRV clade was not in the cell line was estimated by recording the number of times a cell line clone was not basal to the clade on a random sample of 1000 trees extracted from the Bayesian posterior distribution.

Tree shape was assessed using the B_1 _statistic[38], accounting for phylogenetic uncertainty by marginalizing B_1 _across a sample of 1000 posterior trees generated by Mr. Bayes (B_1 _average = 24.27, 95% highest posterior density interval = 20.98-26.75). The null distribution of B_1 _was calculated by simulating 1000 phylogenetic trees with 55 taxa (i.e. the size of the XMRV clade). Significance was assessed by counting the number of times B_1 _values less than the mean B_1 _value occurred in the simulation (2 in 1000 replicates).

The phylogenies of the *gag *(783 nt; n = 6), *pol *(381 nt; n = 9) and *env *(516 nt; n = 9) MLV-X sequence fragments amplified from tumour cell lines were estimated as described above. The sequences were compared to nonecotropic endogenous MLVs (n = 95), AKV (GenBank Acc. J01998) and Moloney MLV (GenBank Acc. AF033811) sequences, as well as 5 XMRV sequences apparently amplified from PC and 2 from CFS samples. The MCMC search was set to 5,000,000 iterations, with trees sampled every 100^th ^generations.

### Recombination analysis

Following visual inspection of the gene sequences derived from PC patient material (n = 15), a 1185 nt *pol *gene fragment from patients VP29 and VP184 was used in a nucleotide BLAST search against all available sequences. Both fragments showed maximal identity with two Moloney MLV complete genomes (GenBank AF033811 and J02255; 99% and 100% identity with VP29 and VP184 respectively). Further evidence of recombination between XMRV and Moloney MLV was sought by examining phylogenetic incongruence in two maximum likelihood trees based on i) the 1185 nt *pol *gene fragment (position 2400 to 3585 of the Moloney MLV AF033811) and ii) the following 1335 nt (position 3586 to 4921). The *pol *sequences used for the analysis comprised 14 patient-derived sequences (see above), one Moloney MLV sequence (GenBank AF033811), one AKV virus sequence (GenBank J01998) and 39 nonecotropic endogenous MLVs[27]. The trees were reconstructed under the GTR+I+G model of evolution, using PAUP*. The robustness of the topologies was assessed by neighbour joining bootstrapping with 1000 replicates.

### Genetic distance analysis

Pairwise nucleotide differences in the *gag *(1605 nt), *pol *(1635 nt) and *env *(1935 nt) of the 22Rv1 and patient-associated sequences were calculated using PAUP*[37]. Genetic distances were estimated i) as the uncorrected number of observed nucleotide substitutions per site and ii) under the GTR+I+G model of evolution. Prior to computation, sequences were screened for APOBEC-3G/F mediated G > A hypermutations, using the Hypermut2.0 algorithm from the Los Alamos HIV Sequence Database[39], and hypermutations masked. A total of 81 and 5 hypermutations were found in the 22Rv1 and patient-associated sequences respectively. The XMRV/Moloney recombinants VP29 and VP184 were excluded. The null hypothesis that genetic diversity is equal in 22Rv1 clones and patient-derived XMRV sequences was tested using the non-parametric Wilcoxon sum rank test.

MLV-X nucleotide sequences obtained from human tumour cell lines have GenBank accession numbers [FR670581-FR670601] and 22Rv1 derived sequences are [HQ385277-HQ385320].

## Competing interests

The authors declare that they have no competing interests.

## Authors' contributions

GJT, SH, PK, JAG, DP conceived the study and SH, ERG, AK, AG, CPT, CJH, SM and AF performed the research. SH, ERG, AK, AG, JAG, OGP, PK & GJT analysed the data and wrote the paper. SH, ERG and AG contributed equally to this work.

## Supplementary Material

Additional file 1 **Table S1: Primers used in this study **. Primers used to non-specifically amplify the *gag*-leader deletion were EG87 and EG89. *Gag*, *pol *and *env *primers were used to amplify sequences from the infected human tumour cell lines (TC primers) or from the 22Rv1 cells (22Rv1 primers). Taqman PCR primer sets used to screen mouse genomic DNA and human tumour cell lines are also shown. Click here for file

Additional file 2 **Table S2: Cancer cell lines screened in this study**. The 411 human tumour cell lines screened by Taqman PCR for MLV-X and XMRV signatures. Detailed are their common name, COSMIC ID, and tumour classification details. No experiments were carried out with 22Rv1 cells until all experiments with tumour cell lines and mouse DNA were completed. NS Not specified. Primers are shown in Table S1. Click here for file

Additional file 3 **Figure S1: Bayesian maximum clade credibility trees based on the *gag *(a), *pol *(b) and *env *(c) loci of known MLV and MLV-X found contaminating human tumour cell lines**. Xenotropic MLV (MLV-X), Polytropic MLV (PMLV) and Modified polytropic MLV (MPMLV) are shown in blue, green and orange circles respectively. XMRVs are represented by blue open circles. MLV-X in the cancer cell lines are indicated in red, the corresponding branch labelled with the cell line name. Bayesian posterior probabilities > 0.90 or 1.00 are indicated on the branches by one or two stars respectively. The scale bar represents the number of nucleotide substitutions per site. Click here for file

Additional file 4 **Figure S2: Maximum likelihood trees showing recombination between XMRV and MoMLV in the sequences derived from PC patients VP29 and VP184**. Between nucleotide positions 2400 and 3585 (GenBank Acc. No. AF033811) of the MoMLV *pol *gene, VP29 and VP184 pol genes are closely related to MoMLV (bootstrap support: 100%) (A), while between positions 3586 to 4921, the same patients derived sequences fall within the XMRV cluster (bootstrap score: 100%) (B). Bootstrap scores above 50% are indicated on the corresponding branches. The scale bar represents the number of nucleotide substitutions per site. Click here for file

Additional file 5 **Figure S3: Bayesian maximum clade credibility phylogeny of 22Rv1 cell line derived XMRV clones, patient derived XMRV sequences and other murine leukaemia viruses based on the (a) *gag*, (b) *pol *and (c) *env *genetic regions only**. Xenotropic MLV (MLV-X), polytropic MLV (PMLV), and modified polytropic MLV (MPMLV) were added as controls. Sequences derived from prostate cancer patients (VP and WO) and chronic fatigue syndrome patients (WPI) are indicated by red and yellow circles respectively. Gene sequences derived from 22Rv1 clones are indicated by blue squares. The trees are rooted by the mid-point rooting method. Bayesian posterior probabilities > 0.95 (*) and of 1.00 (**) are indicated on the corresponding branches. The branching order of the sequences within the XMRV clusters is not statistically supported and therefore cannot be determined unambiguously from these trees. For this reason we have reconstructed a Bayesian phylogeny from the fragments together with the full-length XMRV sequences (Figure 2). The scale bar represents the number of nucleotide substitutions per site. Click here for file

Additional file 6 **Table S3: Genetic diversity of the cell-line and patient-derived *gag*, *pol *and *env *gene sequences**. Genetic distances were calculated as i) the observed number of nucleotide substitutions per sites and ii) under the General Time Reversible model of nucleotide substitutions. The significance of difference in the mean genetic diversity between cell line- and patient-derived sequences was tested by Wilcoxon sum rank test. Click here for file
