# Supplementary material for: Disease-associated XMRV sequences are consistent with laboratory contamination
Source: Retrovirology. 2010 Dec 20;7:111. doi: 10.1186/1742-4690-7-111 (PMC3018392; doi:10.1186/1742-4690-7-111)
Supplement: Additional file 1 — Table S1: Primers used in this study . Primers used to non-specifically amplify the gag-leader deletion were EG87 and EG89. Gag, pol and env primers were used to amplify sequences from the infected human tumour cell lines (TC primers) or from the 22Rv1 cells (22Rv1 primers). Taqman PCR primer sets used to screen mouse genomic DNA and human tumour cell lines are also shown. [file 1742-4690-7-111-S1.DOC]

Table S1. Primers used in this study

| **Primer Name** | **Sequence** |
| --- | --- |
| EG87 | GCTAACTAGATCTGTATCTGGCGG |
| EG89 | CCGAAAGCAAAAATTCAGACGG |
| TC *Gag* F | TCCCCACGGACACCCGGATC |
| TC *Gag* R | GAGGACGGACTCAATCAAGGCC |
| TC *Pol* F | AAGCTAGACCCAGTGGCAGCCG |
| TC *Pol* R | TGCCATCTTTAAGGCTTGGGTGAGTG |
| TC *Env* F | AAGATAAGATTAACCCGTGGGGCC |
| TC *Env* R | CATGTTTTGGGGGCATCCCAGC |
| 22Rv1 *Gag* F | CGAAACCGCGCCGCGCGTCTG |
| 22Rv1 *Gag* R | CCGACTTTGAGGGTTATCCTG |
| 22Rv1 *Pol* F | AAATCCACTTCGAGGGATCAG |
| 22Rv1 *Pol* R | GGTGAGTGCTATCAGTTCGGC |
| 22Rv1 *Env* F | CGCTCACGTAAAGGCGGCGAC |
| 22Rv1 *Env* R | GGCGTTACTGTAGCTAGCGTG |
| XMRV *gag-leader* | F-GGACTTTTTGGAGTGGCTTTGTT  R-GCGTAAAACCGAAAGCAAAAAT  P-FAM-ACAGAGACACTTCCCGCCCCCG-BHQ1 |
| XMRV *integrase* | F-CGAGAGGCAGCCATGAAGG  R-GAGATCTGTTTCGGTGTAATGGAAA  P-FAM-AGTTCTAGAAACCTCTACACTC-MGBNFQ |
| MLV-X *gag* | F-AACCGTTTGTCTCTCCTAAACCC  R-GCAGGGTAAAGGGCAGATCG  P-FAM-ACCGACAGCTCCCGTCCTCCCG-TAMRA |

Primers used to non-specifically amplify the *gag*-leader deletion were EG87 and EG89. *Gag*, *pol* and *env* primers were used to amplify sequences from the infected human tumour cell lines (TC primers) or from the 22Rv1 cells (22Rv1 primers). TaqMan PCR primer sets used to screen mouse genomic DNA and human tumour cell lines are also shown.
