# Supplementary material for: Disease-associated XMRV sequences are consistent with laboratory contamination
Source: Retrovirology. 2010 Dec 20;7:111. doi: 10.1186/1742-4690-7-111 (PMC3018392; doi:10.1186/1742-4690-7-111)
Supplement: Additional file 2 — Table S2: Cancer cell lines screened in this study. The 411 human tumour cell lines screened by Taqman PCR for MLV-X and XMRV signatures. Detailed are their common name, COSMIC ID, and tumour classification details. No experiments were carried out with 22Rv1 cells until all experiments with tumour cell lines and mouse DNA were completed. NS Not specified. Primers are shown in Table S1. [file 1742-4690-7-111-S2.DOC]

**Table S2. Cancer cell lines screened in this study**

| **Cancer cell line** | |  | | | | | |
| --- | --- | --- | --- | --- | --- | --- | --- |
| **Name** | **COSMIC ID** | **Primary site** | **Site subtype 1** | **Site subtype 2** | **Primary Histology** | **Histology subtype 1** | **Histology subtype 2** |
|  |  |  |  |  |  |  |  |
| 23132-87 | 910924 | stomach | NS | NS | carcinoma | adenocarcinoma | NS |
| 5637 | 687452 | urinary tract | bladder | NS | carcinoma | NS | NS |
| 697 | 906800 | haematopoietic and lymphoid tissue | NS | NS | haematopoietic neoplasm | acute lymphoblastic leukaemia | NS |
| 786-0 | 905947 | kidney | NS | NS | carcinoma | clear cell renal cell carcinoma | NS |
| A101D | 910921 | skin | NS | NS | malignant melanoma | NS | NS |
| A2058 | 906792 | skin | NS | NS | malignant melanoma | NS | NS |
| A253 | 906794 | salivary gland | submaxillary | NS | carcinoma | mucoepidermoid carcinoma | NS |
| A2780 | 906804 | ovary | NS | NS | carcinoma | adenocarcinoma | NS |
| A375 | 906793 | skin | NS | NS | malignant melanoma | NS | NS |
| A388 | 910697 | NS | NS | NS | carcinoma | NS | NS |
| A427 | 910851 | lung | NS | NS | carcinoma | NS | NS |
| A431 | 910925 | skin | NS | NS | carcinoma | squamous cell carcinoma | NS |
| A498 | 905948 | kidney | NS | NS | carcinoma | NS | NS |
| A4-Fuk | 910934 | skin | NS | NS | malignant melanoma | NS | NS |
| A549 | 905949 | lung | NS | NS | carcinoma | NS | NS |
| A673 | 684052 | soft tissue | striated muscle | NS | rhabdomyosarcoma | NS | NS |
| A704 | 910920 | kidney | NS | NS | carcinoma | renal cell carcinoma | NS |
| ACHN | 905950 | kidney | NS | NS | carcinoma | renal cell carcinoma | NS |
| AGS | 906790 | stomach | NS | NS | carcinoma | adenocarcinoma | NS |
| ALL-PO | 910944 | haematopoietic and lymphoid tissue | NS | NS | lymphoid neoplasm | acute lymphoblastic leukaemia | NS |
| AM-38 | 910933 | central nervous system | brain | NS | glioma | astrocytoma Grade IV | glioblastoma multiforme |
| AN3-CA | 910781 | endometrium | NS | NS | carcinoma | adenocarcinoma | NS |
| ARH-77 | 906765 | haematopoietic and lymphoid tissue | NS | NS | haematopoietic neoplasm | plasma cell myeloma | NS |
| AsPC-1 | 910702 | pancreas | NS | NS | carcinoma | NS | NS |
| BFTC-905 | 910926 | urinary tract | bladder | NS | carcinoma | transitional cell carcinoma | papillary transitional cell carcinoma |
| BFTC-909 | 910698 | kidney | NS | NS | carcinoma | NS | NS |
| BHT-101 | 906696 | thyroid | NS | NS | carcinoma | anaplastic carcinoma | NS |
| BHY | 753535 | upper aerodigestive tract | head neck | NS | carcinoma | squamous cell carcinoma | NS |
| BOKU | 753536 | cervix | NS | NS | carcinoma | squamous cell carcinoma | NS |
| BPH-1 | 924105 | prostate | NS | NS | hyperplasia | NS | NS |
| BT-20 | 906801 | breast | NS | NS | carcinoma | NS | NS |
| BT-474 | 946359 | breast | NS | NS | carcinoma | ductal carcinoma | NS |
| BT-549 | 905951 | breast | NS | NS | carcinoma | ductal carcinoma | papillary |
| BV-173 | 910710 | haematopoietic and lymphoid tissue | NS | NS | haematopoietic neoplasm | blast phase chronic myeloid leukaemia | NS |
| BxPC-3 | 906693 | pancreas | NS | NS | carcinoma | NS | NS |
| C2BBe1 | 910700 | large intestine | colon | NS | carcinoma | adenocarcinoma | NS |
| C32 | 906830 | skin | NS | NS | malignant melanoma | NS | NS |
| C-4-II | 910553 | cervix | NS | NS | carcinoma | NS | NS |
| Ca9-22 | 753538 | upper aerodigestive tract | mouth | gingiva | carcinoma | squamous cell carcinoma | NS |
| CAKI-1 | 905963 | kidney | NS | NS | carcinoma | clear cell renal cell carcinoma | NS |
| CAL-120 | 906826 | breast | NS | NS | carcinoma | NS | NS |
| CAL-12T | 753540 | lung | NS | NS | carcinoma | non small cell carcinoma | NS |
| CAL-148 | 924106 | breast | NS | NS | carcinoma | ductal carcinoma | NS |
| CAL-27 | 910916 | upper aerodigestive tract | mouth | tongue | carcinoma | squamous cell carcinoma | NS |
| CAL-33 | 753541 | upper aerodigestive tract | mouth | tongue | carcinoma | squamous cell carcinoma | NS |
| CAL-39 | 924107 | vulva | NS | NS | carcinoma | squamous cell carcinoma | NS |
| CAL-51 | 910927 | breast | NS | NS | carcinoma | NS | NS |
| CAL-54 | 910952 | kidney | NS | NS | carcinoma | renal cell carcinoma | NS |
| CAL-62 | 906828 | thyroid | NS | NS | carcinoma | anaplastic carcinoma | NS |
| Calu-1 | 724858 | lung | NS | NS | carcinoma | mucoepidermoid carcinoma | NS |
| Calu-3 | 687777 | lung | NS | NS | carcinoma | adenocarcinoma | NS |
| CAMA-1 | 946382 | breast | NS | NS | carcinoma | NS | NS |
| Caov-3 | 906825 | ovary | NS | NS | carcinoma | adenocarcinoma | NS |
| Caov-4 | 949090 | ovary | NS | NS | carcinoma | adenocarcinoma | NS |
| CAPAN-1 | 753624 | pancreas | NS | NS | carcinoma | ductal carcinoma | NS |
| Capan-2 | 910915 | pancreas | NS | NS | carcinoma | NS | NS |
| CAS-1 | 910943 | central nervous system | brain | NS | glioma | astrocytoma Grade IV | glioblastoma multiforme |
| Ca-Ski | 906824 | cervix | NS | NS | carcinoma | squamous cell carcinoma | NS |
| CCF-STTG1 | 906823 | central nervous system | brain | NS | glioma | astrocytoma Grade IV | glioblastoma multiforme |
| CCRF-CEM | 905952 | haematopoietic and lymphoid tissue | NS | NS | haematopoietic neoplasm | acute lymphoblastic leukaemia | NS |
| CFPAC-1 | 906821 | pancreas | NS | NS | carcinoma | ductal carcinoma | NS |
| CGTH-W-1 | 910568 | thyroid | NS | NS | carcinoma | follicular carcinoma | NS |
| ChaGo-K-1 | 687596 | lung | NS | NS | carcinoma | NS | NS |
| CHL-1 | 910853 | skin | NS | NS | malignant melanoma | NS | NS |
| CHP-134 | 910941 | autonomic ganglia | NS | NS | neuroblastoma | NS | NS |
| CHP-212 | 906820 | autonomic ganglia | NS | NS | neuroblastoma | NS | NS |
| CoCM-1 | 910783 | large intestine | colon | descending | carcinoma | adenocarcinoma | NS |
| COLO-205 | 905961 | large intestine | colon | NS | carcinoma | adenocarcinoma | NS |
| COLO-320-HSR | 910569 | large intestine | colon | NS | carcinoma | adenocarcinoma | NS |
| COLO-668 | 910692 | lung | NS | NS | carcinoma | small cell carcinoma | NS |
| COLO-679 | 906818 | skin | NS | NS | malignant melanoma | NS | NS |
| COLO-684 | 910691 | endometrium | NS | NS | carcinoma | adenocarcinoma | NS |
| COLO-741 | 906815 | large intestine | colon | NS | carcinoma | NS | NS |
| COLO-792 | 906814 | skin | NS | NS | malignant melanoma | NS | NS |
| COLO-800 | 906813 | skin | NS | NS | malignant melanoma | NS | NS |
| COLO-824 | 906812 | breast | NS | NS | carcinoma | NS | NS |
| COR-L105 | 906805 | lung | NS | NS | carcinoma | adenocarcinoma | NS |
| COR-L23 | 687780 | lung | NS | NS | carcinoma | large cell carcinoma | NS |
| COR-L279 | 910937 | lung | NS | NS | carcinoma | small cell carcinoma | NS |
| COR-L88 | 906808 | lung | NS | NS | carcinoma | small cell carcinoma | NS |
| CP67-MEL | 949092 | skin | NS | NS | malignant melanoma | NS | NS |
| CTB-1 | 949088 | haematopoietic and lymphoid tissue | NS | NS | lymphoid neoplasm | B cell lymphoma unspecified | NS |
| CTV-1 | 753548 | haematopoietic and lymphoid tissue | NS | NS | haematopoietic neoplasm | acute myeloid leukaemia | M5 |
| CW-2 | 910554 | large intestine | colon | NS | carcinoma | NS | NS |
| D-247MG | 946367 | central nervous system | brain | NS | glioma | astrocytoma | NS |
| D-283MED | 906834 | central nervous system | NS | NS | primitive neuroectodermal tumour-medulloblastoma | NS | NS |
| D-423MG | 946372 | central nervous system | brain | NS | glioma | astrocytoma | NS |
| Daoy | 906833 | central nervous system | posterior fossa | NS | primitive neuroectodermal tumour-medulloblastoma | desmoplastic | NS |
| Daudi | 906831 | haematopoietic and lymphoid tissue | NS | NS | lymphoid neoplasm | Burkitt lymphoma | NS |
| DB | 906832 | haematopoietic and lymphoid tissue | NS | NS | lymphoid neoplasm | diffuse large B cell lymphoma | NS |
| DK-MG | 906839 | central nervous system | parietal lobe | NS | glioma | astrocytoma Grade IV | glioblastoma multiforme |
| DMS-114 | 687983 | lung | NS | NS | carcinoma | small cell carcinoma | NS |
| DMS-53 | 907295 | lung | NS | NS | carcinoma | small cell carcinoma | NS |
| DMS-79 | 753551 | lung | NS | NS | carcinoma | small cell carcinoma | NS |
| DOHH-2 | 906842 | haematopoietic and lymphoid tissue | NS | NS | lymphoid neoplasm | diffuse large B cell lymphoma | NS |
| DOK | 910936 | upper aerodigestive tract | mouth | tongue | other | epithelial dysplasia | NS |
| DoTc2-4510 | 906843 | cervix | NS | NS | carcinoma | NS | NS |
| DSH1 | 753552 | urinary tract | bladder | NS | carcinoma | NS | NS |
| DU-145 | 905935 | prostate | NS | NS | carcinoma | adenocarcinoma | NS |
| DU-4475 | 906844 | breast | NS | NS | carcinoma | ductal carcinoma | NS |
| ECC10 | 906848 | stomach | NS | NS | carcinoma | small cell adenocarcinoma | NS |
| ECC4 | 906850 | gastrointestinal tract (site indeterminate) | NS | NS | carcinoma | small cell carcinoma | NS |
| EFM-19 | 906851 | breast | NS | NS | carcinoma | ductal carcinoma | NS |
| EFO-21 | 911905 | ovary | NS | NS | carcinoma | serous carcinoma | NS |
| EKVX | 905970 | lung | NS | NS | carcinoma | adenocarcinoma | NS |
| ES1 | 949158 | bone | NS | NS | Ewings sarcoma-peripheral primitive neuroectodermal tumour | NS | NS |
| ES3 | 684055 | bone | NS | NS | Ewings sarcoma-peripheral primitive neuroectodermal tumour | NS | NS |
| ES4 | 949156 | bone | NS | NS | Ewings sarcoma-peripheral primitive neuroectodermal tumour | NS | NS |
| ES5 | 684057 | bone | NS | NS | Ewings sarcoma-peripheral primitive neuroectodermal tumour | NS | NS |
| EW-1 | 949163 | bone | NS | NS | Ewings sarcoma-peripheral primitive neuroectodermal tumour | NS | NS |
| EW-11 | 684062 | bone | NS | NS | Ewings sarcoma-peripheral primitive neuroectodermal tumour | NS | NS |
| EW-12 | 949162 | bone | NS | NS | Ewings sarcoma-peripheral primitive neuroectodermal tumour | NS | NS |
| EW-13 | 949166 | bone | NS | NS | Ewings sarcoma-peripheral primitive neuroectodermal tumour | NS | NS |
| EW-16 | 949165 | bone | NS | NS | Ewings sarcoma-peripheral primitive neuroectodermal tumour | NS | NS |
| EW-18 | 949164 | bone | NS | NS | Ewings sarcoma-peripheral primitive neuroectodermal tumour | NS | NS |
| EW-22 | 949167 | bone | NS | NS | Ewings sarcoma-peripheral primitive neuroectodermal tumour | NS | NS |
| EW-24 | 949168 | bone | NS | NS | Ewings sarcoma-peripheral primitive neuroectodermal tumour | NS | NS |
| EW-3 | 949161 | bone | NS | NS | Ewings sarcoma-peripheral primitive neuroectodermal tumour | NS | NS |
| EW-7 | 949160 | bone | NS | NS | Ewings sarcoma-peripheral primitive neuroectodermal tumour | NS | NS |
| FADU | 906863 | upper aerodigestive tract | pharynx | hypopharynx | carcinoma | squamous cell carcinoma | NS |
| G-361 | 906865 | skin | NS | NS | malignant melanoma | NS | NS |
| G-401 | 907299 | kidney | NS | NS | rhabdoid tumour | NS | NS |
| G-402 | 907298 | soft tissue | smooth muscle | kidney | leiomyoblastoma | NS | NS |
| GA-10-Clone-20 | 906866 | haematopoietic and lymphoid tissue | NS | NS | lymphoid neoplasm | Burkitt lymphoma | NS |
| GAK | 910932 | skin | NS | NS | malignant melanoma | NS | NS |
| GCIY | 906869 | stomach | NS | NS | carcinoma | adenocarcinoma | NS |
| GR-ST | 906877 | haematopoietic and lymphoid tissue | NS | NS | haematopoietic neoplasm | acute lymphoblastic leukaemia | NS |
| H9 | 907043 | haematopoietic and lymphoid tissue | skin | NS | lymphoid neoplasm | mycosis fungoides-Sezary syndrome | NS |
| HAL-01 | 949153 | haematopoietic and lymphoid tissue | NS | NS | lymphoid neoplasm | acute lymphoblastic leukaemia | NS |
| HCC1419 | 907045 | breast | NS | NS | carcinoma | ductal carcinoma | NS |
| HCC1569 | 907046 | breast | NS | NS | carcinoma | NS | NS |
| HCC2998 | 905971 | large intestine | colon | NS | carcinoma | adenocarcinoma | NS |
| HCT-116 | 905936 | large intestine | colon | NS | carcinoma | NS | NS |
| HCT-15 | 905937 | large intestine | colon | NS | carcinoma | NS | NS |
| HDLM-2 | 924110 | haematopoietic and lymphoid tissue | lymph node | NS | lymphoid neoplasm | Hodgkin lymphoma | NS |
| HD-MY-Z | 907050 | haematopoietic and lymphoid tissue | lymph node | NS | lymphoid neoplasm | Hodgkin lymphoma | NS |
| HEC-1 | 907051 | endometrium | NS | NS | carcinoma | adenocarcinoma | NS |
| HeLaSF | 687509 | cervix | NS | NS | carcinoma | adenocarcinoma | NS |
| H-EMC-SS | 907290 | bone | extraskeletal | NS | chondrosarcoma | NS | NS |
| HGC-27 | 907055 | stomach | NS | NS | carcinoma | NS | NS |
| HL-60 | 905938 | haematopoietic and lymphoid tissue | NS | NS | haematopoietic neoplasm | acute myeloid leukaemia | M3 |
| HLE | 907057 | liver | NS | NS | carcinoma | hepatocellular carcinoma | NS |
| HMV-II | 907058 | skin | NS | NS | malignant melanoma | NS | NS |
| HN | 907059 | upper aerodigestive tract | mouth | mouth roof | carcinoma | squamous cell carcinoma | NS |
| HOP-62 | 905972 | lung | NS | NS | carcinoma | adenocarcinoma | NS |
| HOP-92 | 905973 | lung | NS | NS | carcinoma | large cell carcinoma | NS |
| HPAF-II | 724869 | pancreas | NS | NS | carcinoma | ductal carcinoma | NS |
| Hs-578-T | 905957 | breast | NS | NS | carcinoma | NS | NS |
| HSC-2 | 753562 | upper aerodigestive tract | mouth | NS | carcinoma | squamous cell carcinoma | NS |
| HT-1080 | 907064 | soft tissue | fibrous tissue and uncertain origin | NS | fibrosarcoma | NS | NS |
| HT-1197 | 907065 | urinary tract | bladder | NS | carcinoma | transitional cell carcinoma | NS |
| HT-144 | 907067 | skin | NS | NS | malignant melanoma | NS | NS |
| HT-29 | 905939 | large intestine | colon | NS | carcinoma | NS | NS |
| HT-3 | 907068 | cervix | NS | NS | carcinoma | NS | NS |
| HT55 | 907287 | large intestine | colon | NS | carcinoma | adenocarcinoma | NS |
| HuH-28 | 924163 | biliary tract | bile duct | NS | carcinoma | NS | NS |
| HUH-6-clone5 | 907070 | liver | NS | NS | carcinoma | hepatocellular carcinoma | NS |
| HuO-3N1 | 909977 | bone | NS | NS | osteosarcoma | NS | NS |
| HuO9 | 907072 | bone | femur | NS | osteosarcoma | NS | NS |
| HuP-T4 | 907286 | pancreas | NS | NS | carcinoma | NS | NS |
| IA-LM | 910779 | lung | NS | NS | carcinoma | large cell carcinoma | NS |
| IGROV-1 | 905968 | ovary | NS | NS | carcinoma | NS | NS |
| IM-9 | 753563 | haematopoietic and lymphoid tissue | NS | NS | lymphoid neoplasm | plasma cell myeloma | NS |
| IMR-5 | 907170 | autonomic ganglia | NS | NS | neuroblastoma | NS | NS |
| IPC-298 | 907171 | skin | NS | NS | malignant melanoma | NS | NS |
| IST-MES1 | 907173 | pleura | NS | NS | mesothelioma | NS | NS |
| IST-SL2 | 753565 | lung | NS | NS | carcinoma | small cell carcinoma | NS |
| J-RT3-T3-5 | 907391 | haematopoietic and lymphoid tissue | NS | NS | lymphoid neoplasm | acute lymphoblastic T cell leukaemia | NS |
| K-562 | 905940 | haematopoietic and lymphoid tissue | NS | NS | haematopoietic neoplasm | chronic myeloid leukaemia | NS |
| KALS-1 | 907271 | central nervous system | brain | NS | glioma | NS | NS |
| KG-1 | 907278 | haematopoietic and lymphoid tissue | NS | NS | haematopoietic neoplasm | acute myeloid leukaemia | M6 |
| KM12 | 905989 | large intestine | colon | NS | carcinoma | adenocarcinoma | NS |
| KM-H2 | 909976 | haematopoietic and lymphoid tissue | lymph node | NS | lymphoid neoplasm | Hodgkin lymphoma | NS |
| KNS-81-FD | 924188 | central nervous system | brain | NS | glioma | NS | NS |
| KU-19-19 | 907312 | urinary tract | bladder | NS | carcinoma | transitional cell carcinoma | NS |
| KU812 | 907311 | haematopoietic and lymphoid tissue | NS | NS | haematopoietic neoplasm | chronic myeloid leukaemia | Ph positive |
| KURAMOCHI | 909975 | ovary | NS | NS | carcinoma | undifferentiated carcinoma | NS |
| KYSE-140 | 753573 | oesophagus | middle third | NS | carcinoma | squamous cell carcinoma | NS |
| KYSE-150 | 907317 | oesophagus | upper third | NS | carcinoma | squamous cell carcinoma | NS |
| KYSE-180 | 907318 | oesophagus | middle third | NS | carcinoma | squamous cell carcinoma | NS |
| KYSE-410 | 753574 | oesophagus | upper third | NS | carcinoma | squamous cell carcinoma | NS |
| KYSE-450 | 907320 | oesophagus | middle third | NS | carcinoma | squamous cell carcinoma | NS |
| KYSE-520 | 753575 | oesophagus | lower third | NS | carcinoma | squamous cell carcinoma | NS |
| L-363 | 924239 | haematopoietic and lymphoid tissue | NS | NS | lymphoid neoplasm | plasma cell myeloma | NS |
| L-428 | 907322 | haematopoietic and lymphoid tissue | lymph node | NS | lymphoid neoplasm | Hodgkin lymphoma | NS |
| LAMA-84 | 907783 | haematopoietic and lymphoid tissue | NS | NS | haematopoietic neoplasm | blast phase chronic myeloid leukaemia | NS |
| LAN-6 | 949170 | autonomic ganglia | NS | NS | neuroblastoma | NS | NS |
| LCLC-103H | 753586 | lung | NS | NS | carcinoma | large cell carcinoma | NS |
| LK-2 | 687787 | lung | NS | NS | carcinoma | squamous cell carcinoma | NS |
| LN-405 | 910694 | central nervous system | brain | NS | glioma | astrocytoma Grade IV | glioblastoma multiforme |
| LOUCY | 907789 | haematopoietic and lymphoid tissue | NS | NS | lymphoid neoplasm | acute lymphoblastic T cell leukaemia | NS |
| LoVo | 907790 | large intestine | colon | NS | carcinoma | adenocarcinoma | NS |
| LOXIMVI | 905974 | skin | NS | NS | malignant melanoma | NS | NS |
| LS-123 | 907792 | large intestine | colon | NS | carcinoma | adenocarcinoma | NS |
| LS-174T | 907793 | large intestine | colon | NS | carcinoma | adenocarcinoma | NS |
| LS-411N | 907794 | large intestine | caecum | NS | carcinoma | adenocarcinoma | NS |
| LS-513 | 907795 | large intestine | caecum | NS | carcinoma | adenocarcinoma | NS |
| LU-134-A | 753588 | lung | NS | NS | carcinoma | small cell carcinoma | NS |
| LU-135 | 713899 | lung | NS | NS | carcinoma | small cell carcinoma | NS |
| LU-65 | 724863 | lung | NS | NS | carcinoma | giant cell carcinoma | NS |
| M059J | 949094 | central nervous system | brain | NS | glioma | astrocytoma Grade IV | glioblastoma multiforme |
| M14 | 905975 | skin | NS | NS | malignant melanoma | NS | NS |
| Malme-3M | 905953 | skin | NS | NS | malignant melanoma | NS | NS |
| MC116 | 907799 | haematopoietic and lymphoid tissue | NS | NS | lymphoid neoplasm | B cell lymphoma unspecified | NS |
| MC-CAR | 683665 | haematopoietic and lymphoid tissue | NS | NS | lymphoid neoplasm | plasmacytoma | NS |
| MCF7 | 905946 | breast | NS | NS | carcinoma | NS | NS |
| MDA-MB-157 | 925338 | breast | NS | NS | carcinoma | ductal carcinoma | medullary |
| MDA-MB-175-VII | 908120 | breast | NS | NS | carcinoma | ductal carcinoma | NS |
| MDA-MB-231 | 905960 | breast | NS | NS | carcinoma | NS | NS |
| MDA-MB-361 | 908121 | breast | NS | NS | carcinoma | NS | NS |
| MDA-MB-415 | 924240 | breast | NS | NS | carcinoma | NS | NS |
| MDA-MB-435 | 905988 | breast | NS | NS | carcinoma | NS | NS |
| MDA-MB-453 | 908122 | breast | NS | NS | carcinoma | NS | NS |
| MDA-MB-468 | 908123 | breast | NS | NS | carcinoma | NS | NS |
| ME-180 | 687514 | cervix | NS | NS | carcinoma | NS | NS |
| MES-SA | 908127 | soft tissue | fibrous tissue and uncertain origin | uterus | sarcoma | NS | NS |
| MFM-223 | 910948 | breast | NS | NS | carcinoma | ductal carcinoma | NS |
| MG-63 | 908131 | bone | NS | NS | osteosarcoma | NS | NS |
| MHH-ES-1 | 908134 | bone | pelvis | NS | Ewings sarcoma-peripheral primitive neuroectodermal tumour | NS | NS |
| MIA-PaCa-2 | 724870 | pancreas | NS | NS | carcinoma | NS | NS |
| MKN1 | 908138 | stomach | NS | NS | carcinoma | NS | NS |
| MKN28 | 908139 | stomach | NS | NS | carcinoma | adenocarcinoma | NS |
| ML-2 | 908141 | haematopoietic and lymphoid tissue | NS | NS | haematopoietic neoplasm | acute myeloid leukaemia | M4 |
| MLMA | 908142 | haematopoietic and lymphoid tissue | NS | NS | lymphoid neoplasm | hairy cell leukaemia | NS |
| MOG-G-CCM | 908144 | central nervous system | brain | NS | glioma | astrocytoma | NS |
| MOLT-4 | 905958 | haematopoietic and lymphoid tissue | NS | NS | haematopoietic neoplasm | acute lymphoblastic leukaemia | NS |
| MONO-MAC-6 | 908148 | haematopoietic and lymphoid tissue | NS | NS | haematopoietic neoplasm | acute myeloid leukaemia | M5 |
| MPP-89 | 908150 | pleura | NS | NS | mesothelioma | NS | NS |
| MUTZ-1 | 908155 | haematopoietic and lymphoid tissue | NS | NS | haematopoietic neoplasm | myelodysplastic syndrome | refractory anaemia with excess blasts |
| MZ2-MEL. | 971777 | skin | NS | NS | malignant melanoma | NS | NS |
| NALM-6 | 908158 | haematopoietic and lymphoid tissue | NS | NS | lymphoid neoplasm | acute lymphoblastic B cell leukaemia | NS |
| NB1 | 949179 | autonomic ganglia | NS | NS | neuroblastoma | NS | NS |
| NB10 | 949171 | autonomic ganglia | NS | NS | neuroblastoma | NS | NS |
| NB13 | 949177 | autonomic ganglia | NS | NS | neuroblastoma | NS | NS |
| NB14 | 949178 | autonomic ganglia | NS | NS | neuroblastoma | NS | NS |
| NB16 | 910929 | autonomic ganglia | NS | NS | neuroblastoma | NS | NS |
| NB17 | 949175 | autonomic ganglia | NS | NS | neuroblastoma | NS | NS |
| NB4 | 908161 | haematopoietic and lymphoid tissue | NS | NS | haematopoietic neoplasm | acute myeloid leukaemia | M3 |
| NB6 | 949173 | autonomic ganglia | NS | NS | neuroblastoma | NS | NS |
| NB69 | 908440 | autonomic ganglia | NS | NS | neuroblastoma | NS | NS |
| NCI-ADR-RES | 905987 | breast | NS | NS | carcinoma | NS | NS |
| NCI-H1048 | 687995 | lung | NS | NS | carcinoma | small cell carcinoma | NS |
| NCI-H1092 | 687997 | lung | NS | NS | carcinoma | small cell carcinoma | NS |
| NCI-H1105 | 908468 | lung | NS | NS | carcinoma | small cell carcinoma | NS |
| NCI-H1155 | 908467 | lung | NS | NS | carcinoma | large cell carcinoma | NS |
| NCI-H1299 | 724831 | lung | NS | NS | carcinoma | large cell carcinoma | NS |
| NCI-H1304 | 753599 | lung | NS | NS | carcinoma | small cell carcinoma | NS |
| NCI-H1355 | 724866 | lung | NS | NS | carcinoma | adenocarcinoma | NS |
| NCI-H146 | 910899 | lung | NS | NS | carcinoma | small cell carcinoma | NS |
| NCI-H1522 | 908464 | lung | NS | NS | carcinoma | small cell carcinoma | NS |
| NCI-H1563 | 753600 | lung | NS | NS | carcinoma | adenocarcinoma | NS |
| NCI-H157 | 911847 | lung | NS | NS | carcinoma | squamous cell carcinoma | NS |
| NCI-H1618 | 753601 | lung | NS | NS | carcinoma | small cell carcinoma | NS |
| NCI-H1623 | 687798 | lung | NS | NS | carcinoma | adenocarcinoma | NS |
| NCI-H1648 | 687799 | lung | NS | NS | carcinoma | adenocarcinoma | NS |
| NCI-H1650 | 687800 | lung | NS | NS | carcinoma | bronchioloalveolar adenocarcinoma | NS |
| NCI-H1666 | 908473 | lung | NS | NS | carcinoma | bronchioloalveolar adenocarcinoma | NS |
| NCI-H1693 | 687802 | lung | NS | NS | carcinoma | adenocarcinoma | NS |
| NCI-H1792 | 724868 | lung | NS | NS | carcinoma | adenocarcinoma | NS |
| NCI-H1793 | 908463 | lung | NS | NS | carcinoma | adenocarcinoma | NS |
| NCI-H1838 | 687807 | lung | NS | NS | carcinoma | adenocarcinoma | NS |
| NCI-H1882 | 753602 | lung | NS | NS | carcinoma | small cell carcinoma | NS |
| NCI-H1926 | 908477 | lung | NS | NS | carcinoma | small cell carcinoma | NS |
| NCI-H1930 | 924245 | lung | NS | NS | carcinoma | small cell carcinoma | NS |
| NCI-H2029 | 688011 | lung | NS | NS | carcinoma | small cell carcinoma | NS |
| NCI-H2030 | 722045 | lung | NS | NS | carcinoma | adenocarcinoma | NS |
| NCI-H2107 | 924246 | lung | NS | NS | carcinoma | small cell carcinoma | NS |
| NCI-H2141 | 688014 | lung | NS | NS | carcinoma | small cell carcinoma | NS |
| NCI-H2196 | 908481 | lung | NS | NS | carcinoma | small cell carcinoma | NS |
| NCI-H2227 | 688018 | lung | NS | NS | carcinoma | small cell carcinoma | NS |
| NCI-H226 | 905941 | lung | NS | NS | carcinoma | squamous cell carcinoma | NS |
| NCI-H23 | 905942 | lung | NS | NS | carcinoma | non small cell carcinoma | NS |
| NCI-H2330 | 688019 | lung | NS | NS | carcinoma | small cell carcinoma | NS |
| NCI-H2452 | 908462 | pleura | NS | NS | mesothelioma | NS | NS |
| NCI-H28 | 908470 | pleura | NS | NS | mesothelioma | NS | NS |
| NCI-H292 | 753604 | lung | NS | NS | carcinoma | mucoepidermoid carcinoma | NS |
| NCI-H295 | 908466 | adrenal gland | adrenal gland | NS | adrenal cortical carcinoma | NS | NS |
| NCI-H322M | 905967 | lung | NS | NS | carcinoma | bronchioloalveolar adenocarcinoma | NS |
| NCI-H345 | 688021 | lung | NS | NS | carcinoma | small cell carcinoma | NS |
| NCI-H358 | 908465 | lung | NS | NS | carcinoma | bronchioloalveolar adenocarcinoma | NS |
| NCI-H378 | 688022 | lung | NS | NS | carcinoma | small cell carcinoma | NS |
| NCI-H441 | 908460 | lung | NS | NS | carcinoma | bronchioloalveolar adenocarcinoma | NS |
| NCI-H446 | 688023 | lung | NS | NS | carcinoma | small cell carcinoma | NS |
| NCI-H460 | 905943 | lung | NS | NS | carcinoma | large cell carcinoma | NS |
| NCI-H508 | 908442 | large intestine | caecum | NS | carcinoma | adenocarcinoma | NS |
| NCI-H510A | 753605 | lung | NS | NS | carcinoma | small cell carcinoma | NS |
| NCI-H520 | 908443 | lung | NS | NS | carcinoma | squamous cell carcinoma | NS |
| NCI-H522 | 905944 | lung | NS | NS | carcinoma | non small cell carcinoma | NS |
| NCI-H526 | 688025 | lung | NS | NS | carcinoma | small cell carcinoma | NS |
| NCI-H64 | 688026 | lung | NS | NS | carcinoma | small cell carcinoma | NS |
| NCI-H650 | 722066 | lung | NS | NS | carcinoma | bronchioloalveolar adenocarcinoma | NS |
| NCI-H661 | 687829 | lung | NS | NS | carcinoma | large cell carcinoma | NS |
| NCI-H69 | 688027 | lung | NS | NS | carcinoma | small cell carcinoma | NS |
| NCI-H711 | 910709 | lung | NS | NS | carcinoma | small cell carcinoma | NS |
| NCI-H719 | 924242 | lung | NS | NS | carcinoma | small cell carcinoma | NS |
| NCI-H720 | 687600 | lung | NS | NS | carcinoid-endocrine tumour | atypical | NS |
| NCI-H748 | 909194 | lung | NS | NS | carcinoma | small cell carcinoma | NS |
| NCI-H810 | 925341 | lung | NS | NS | carcinoma | large cell carcinoma | NS |
| NCI-H82 | 688031 | lung | NS | NS | carcinoma | small cell carcinoma | NS |
| NCI-H838 | 910399 | lung | NS | NS | carcinoma | non small cell carcinoma | NS |
| NCI-H889 | 908456 | lung | NS | NS | carcinoma | small cell carcinoma | NS |
| NCI-SNU-5 | 908445 | stomach | NS | NS | carcinoma | NS | NS |
| NCI-SR | 905965 | haematopoietic and lymphoid tissue | NS | NS | lymphoid neoplasm | NS | NS |
| NEC8 | 910942 | testis | NS | NS | germ cell tumour | embryonal carcinoma | NS |
| NMC-G1 | 908449 | central nervous system | brain | NS | glioma | NS | NS |
| no-11 | 908450 | central nervous system | brain | NS | glioma | astrocytoma Grade III | anaplastic |
| NOMO-1 | 908451 | haematopoietic and lymphoid tissue | NS | NS | haematopoietic neoplasm | acute myeloid leukaemia | M5 |
| NUGC-3 | 908455 | stomach | NS | NS | carcinoma | NS | NS |
| NY | 910849 | bone | NS | NS | osteosarcoma | NS | NS |
| OAW-42 | 910548 | ovary | NS | NS | carcinoma | mucinous carcinoma | NS |
| OE19 | 910079 | oesophagus | lower third | NS | carcinoma | adenocarcinoma | NS |
| OE33 | 910549 | oesophagus | lower third | NS | other | metaplasia | NS |
| OMC-1 | 949154 | cervix | NS | NS | carcinoma | squamous cell carcinoma | NS |
| OS-RC-2 | 909250 | kidney | NS | NS | carcinoma | renal cell carcinoma | NS |
| OVCAR-3 | 905933 | ovary | NS | NS | carcinoma | adenocarcinoma | NS |
| OVCAR-4 | 905990 | ovary | NS | NS | carcinoma | NS | NS |
| OVCAR-5 | 905969 | ovary | NS | NS | carcinoma | NS | NS |
| OVCAR-8 | 905991 | ovary | NS | NS | carcinoma | NS | NS |
| PC-14 | 753608 | lung | NS | NS | carcinoma | adenocarcinoma | NS |
| PC3 | 850414 | prostate | NS | NS | carcinoma | adenocarcinoma | NS |
| PSN1 | 910546 | pancreas | NS | NS | carcinoma | NS | NS |
| QIMR-WIL | 910545 | haematopoietic and lymphoid tissue | NS | NS | haematopoietic neoplasm | acute myeloid leukaemia | NS |
| RCC10RGB | 909974 | kidney | NS | NS | carcinoma | renal cell carcinoma | NS |
| REH | 909696 | haematopoietic and lymphoid tissue | NS | NS | lymphoid neoplasm | acute lymphoblastic B cell leukaemia | NS |
| RF-48 | 909697 | stomach | NS | NS | carcinoma | adenocarcinoma | NS |
| RH-1 | 971773 | soft tissue | striated muscle | NS | rhabdomyosarcoma | NS | NS |
| RH-18 | 971774 | soft tissue | striated muscle | abdomen | rhabdomyosarcoma | embryonal | NS |
| RKO | 909698 | large intestine | colon | NS | carcinoma | NS | NS |
| RL | 910861 | haematopoietic and lymphoid tissue | NS | NS | lymphoid neoplasm | B cell lymphoma unspecified | NS |
| RPMI-2650 | 909700 | upper aerodigestive tract | sinonasal and nasal cavity | NS | carcinoma | squamous cell carcinoma | NS |
| RPMI-8226 | 905964 | haematopoietic and lymphoid tissue | NS | NS | haematopoietic neoplasm | plasma cell myeloma | NS |
| RT-112 | 909704 | urinary tract | bladder | NS | carcinoma | transitional cell carcinoma | NS |
| RXF393 | 905978 | kidney | NS | NS | carcinoma | renal cell carcinoma | NS |
| S-117 | 910946 | soft tissue | fibrous tissue and uncertain origin | thyroid | sarcoma | NS | NS |
| Saos-2 | 909707 | bone | NS | NS | osteosarcoma | NS | NS |
| SAS | 909708 | upper aerodigestive tract | mouth | tongue | carcinoma | squamous cell carcinoma | NS |
| SCC-15 | 910911 | upper aerodigestive tract | mouth | tongue | carcinoma | squamous cell carcinoma | NS |
| SCC-25 | 910701 | upper aerodigestive tract | mouth | tongue | carcinoma | squamous cell carcinoma | NS |
| SCC-4 | 910904 | upper aerodigestive tract | mouth | tongue | carcinoma | squamous cell carcinoma | NS |
| SF268 | 905986 | central nervous system | brain | NS | glioma | NS | NS |
| SF295 | 905985 | central nervous system | brain | NS | glioma | NS | NS |
| SF539 | 905984 | central nervous system | brain | NS | glioma | NS | NS |
| SHP-77 | 724872 | lung | left upper lobe | NS | carcinoma | small cell carcinoma | NS |
| SiHa | 930297 | cervix | NS | NS | carcinoma | squamous cell carcinoma | NS |
| SIMA | 753620 | autonomic ganglia | NS | NS | neuroblastoma | NS | NS |
| SKG-IIIa | 930298 | cervix | NS | NS | carcinoma | NS | NS |
| SK-LU-1 | 909721 | lung | NS | NS | carcinoma | adenocarcinoma | NS |
| SK-MEL-1 | 909723 | skin | NS | NS | malignant melanoma | NS | NS |
| SK-MEL-2 | 905955 | skin | upper leg | NS | malignant melanoma | NS | NS |
| SK-MEL-24 | 909725 | skin | NS | NS | malignant melanoma | NS | NS |
| SK-MEL-28 | 905954 | skin | NS | NS | malignant melanoma | NS | NS |
| SK-MEL-3 | 909724 | skin | NS | NS | malignant melanoma | NS | NS |
| SK-MEL-5 | 905956 | skin | axilla | NS | malignant melanoma | NS | NS |
| SK-MES-1 | 909728 | lung | NS | NS | carcinoma | squamous cell carcinoma | NS |
| SK-N-DZ | 688086 | autonomic ganglia | NS | NS | neuroblastoma | NS | NS |
| SK-OV-3 | 905959 | ovary | NS | NS | carcinoma | adenocarcinoma | NS |
| SK-UT-1 | 909732 | soft tissue | smooth muscle | uterus | leiomyosarcoma | NS | NS |
| SN12C | 905979 | kidney | NS | NS | carcinoma | renal cell carcinoma | NS |
| SNB19 | 905966 | central nervous system | cerebrum | NS | glioma | astrocytoma Grade IV | glioblastoma multiforme |
| SNB75 | 905982 | central nervous system | brain | NS | glioma | NS | NS |
| SNG-M | 909735 | endometrium | NS | NS | carcinoma | adenocarcinoma | NS |
| SNU-423 | 909737 | liver | NS | NS | carcinoma | hepatocellular carcinoma | NS |
| SNU-475 | 909739 | liver | NS | NS | carcinoma | hepatocellular carcinoma | NS |
| SNU-C1 | 910905 | large intestine | colon | NS | carcinoma | adenocarcinoma | NS |
| ST486 | 910906 | haematopoietic and lymphoid tissue | NS | NS | lymphoid neoplasm | Burkitt lymphoma | NS |
| SW1088 | 909745 | central nervous system | brain | NS | glioma | astrocytoma | NS |
| SW1116 | 909746 | large intestine | colon | NS | carcinoma | adenocarcinoma | NS |
| SW13 | 909744 | adrenal gland | adrenal gland | NS | adrenal cortical carcinoma | NS | NS |
| SW1463 | 909748 | large intestine | rectum | NS | carcinoma | adenocarcinoma | NS |
| SW1710 | 909749 | urinary tract | bladder | NS | carcinoma | transitional cell carcinoma | NS |
| SW1783 | 909750 | central nervous system | brain | NS | glioma | astrocytoma Grade III | anaplastic |
| SW1990 | 910907 | pancreas | NS | NS | carcinoma | NS | NS |
| SW403 | 910908 | large intestine | colon | NS | carcinoma | adenocarcinoma | NS |
| SW620 | 905962 | large intestine | colon | NS | carcinoma | adenocarcinoma | NS |
| SW626 | 909753 | ovary | NS | NS | carcinoma | adenocarcinoma | NS |
| SW684 | 909754 | soft tissue | fibrous tissue and uncertain origin | NS | fibrosarcoma | NS | NS |
| SW756 | 724839 | cervix | NS | NS | carcinoma | squamous cell carcinoma | NS |
| SW872 | 909756 | soft tissue | fat | NS | liposarcoma | NS | NS |
| SW962 | 909758 | vulva | NS | NS | carcinoma | squamous cell carcinoma | NS |
| SW982 | 909759 | soft tissue | fibrous tissue and uncertain origin | NS | synovial sarcoma | NS | NS |
| T47D | 905945 | breast | NS | NS | carcinoma | ductal carcinoma | NS |
| TCCSUP | 687459 | urinary tract | bladder | NS | carcinoma | transitional cell carcinoma | NS |
| TCO-1 | 909763 | thyroid | NS | NS | carcinoma | NS | NS |
| TE-161-T | 909765 | haematopoietic and lymphoid tissue | lymph node | NS | lymphoid neoplasm | Burkitt lymphoma | NS |
| TGBC24TKB | 910695 | biliary tract | bile duct | NS | carcinoma | NS | NS |
| TGW | 910780 | autonomic ganglia | NS | NS | neuroblastoma | NS | NS |
| THP-1 | 909771 | haematopoietic and lymphoid tissue | NS | NS | haematopoietic neoplasm | acute myeloid leukaemia | M5 |
| TI-73 | 910696 | bone | NS | NS | osteosarcoma | NS | NS |
| TK10 | 905980 | kidney | NS | NS | carcinoma | renal cell carcinoma | NS |
| TT | 930299 | thyroid | NS | NS | carcinoma | medullary carcinoma | NS |
| TUR | 909773 | haematopoietic and lymphoid tissue | NS | NS | lymphoid neoplasm | B cell lymphoma unspecified | NS |
| TYK-nu | 909774 | ovary | NS | NS | carcinoma | undifferentiated carcinoma | NS |
| U031 | 905981 | kidney | NS | NS | carcinoma | renal cell carcinoma | NS |
| U-118-MG | 687588 | central nervous system | brain | NS | glioma | astrocytoma Grade IV | glioblastoma multiforme |
| U251 | 905983 | central nervous system | brain | NS | glioma | NS | NS |
| U-266 | 753615 | haematopoietic and lymphoid tissue | NS | NS | lymphoid neoplasm | plasma cell myeloma | NS |
| U-2-OS | 909776 | bone | tibia | NS | osteosarcoma | NS | NS |
| U-698-M | 909777 | haematopoietic and lymphoid tissue | NS | NS | lymphoid neoplasm | acute lymphoblastic B cell leukaemia | NS |
| U-87-MG | 687590 | central nervous system | brain | NS | glioma | astrocytoma Grade IV | glioblastoma multiforme |
| UACC-257 | 905977 | skin | NS | NS | malignant melanoma | NS | NS |
| UACC-62 | 905976 | skin | NS | NS | malignant melanoma | NS | NS |
| UACC-812 | 910910 | breast | NS | NS | carcinoma | ductal carcinoma | NS |
| UACC-893 | 909778 | breast | NS | NS | carcinoma | ductal carcinoma | NS |
| UMC-11 | 909779 | lung | NS | NS | carcinoid-endocrine tumour | NS | NS |
| UM-UC-3 | 724838 | urinary tract | bladder | NS | carcinoma | transitional cell carcinoma | NS |
| VM-CUB-1 | 909780 | urinary tract | bladder | NS | carcinoma | transitional cell carcinoma | NS |
| VMRC-MELG | 930301 | skin | NS | NS | malignant melanoma | NS | NS |
| VMRC-RCZ | 909781 | kidney | NS | NS | carcinoma | renal cell carcinoma | NS |
| WM-115 | 909784 | skin | NS | NS | malignant melanoma | NS | NS |
| YH-13 | 909905 | central nervous system | brain | NS | glioma | astrocytoma Grade IV | glioblastoma multiforme |
| YKG-1 | 687592 | central nervous system | temporal lobe | NS | glioma | astrocytoma Grade IV | glioblastoma multiforme |

The 411 human tumour cell lines screened by Taqman PCR for MLV-X and XMRV signatures. Detailed are their common name, COSMIC ID, and tumour classification details. No experiments were carried out with 22Rv1 cells until all experiments with tumour cell lines and mouse DNA were completed. NS Not specified. Primers are shown in Table S1.
