# Supplementary material for: Disease-associated XMRV sequences are consistent with laboratory contamination
Source: Retrovirology. 2010 Dec 20;7:111. doi: 10.1186/1742-4690-7-111 (PMC3018392; doi:10.1186/1742-4690-7-111)
Supplement: Additional file 3 — Figure S1: Bayesian maximum clade credibility trees based on the gag (a), pol (b) and env (c) loci of known MLV and MLV-X found contaminating human tumour cell lines. Xenotropic MLV (MLV-X), Polytropic MLV (PMLV) and Modified polytropic MLV (MPMLV) are shown in blue, green and orange circles respectively. XMRVs are represented by blue open circles. MLV-X in the cancer cell lines are indicated in red, the corresponding branch labelled with the cell line name. Bayesian posterior probabilities > 0.90 or 1.00 are indicated on the branches by one or two stars respectively. The scale bar represents the number of nucleotide substitutions per site. [file 1742-4690-7-111-S3.PDF]

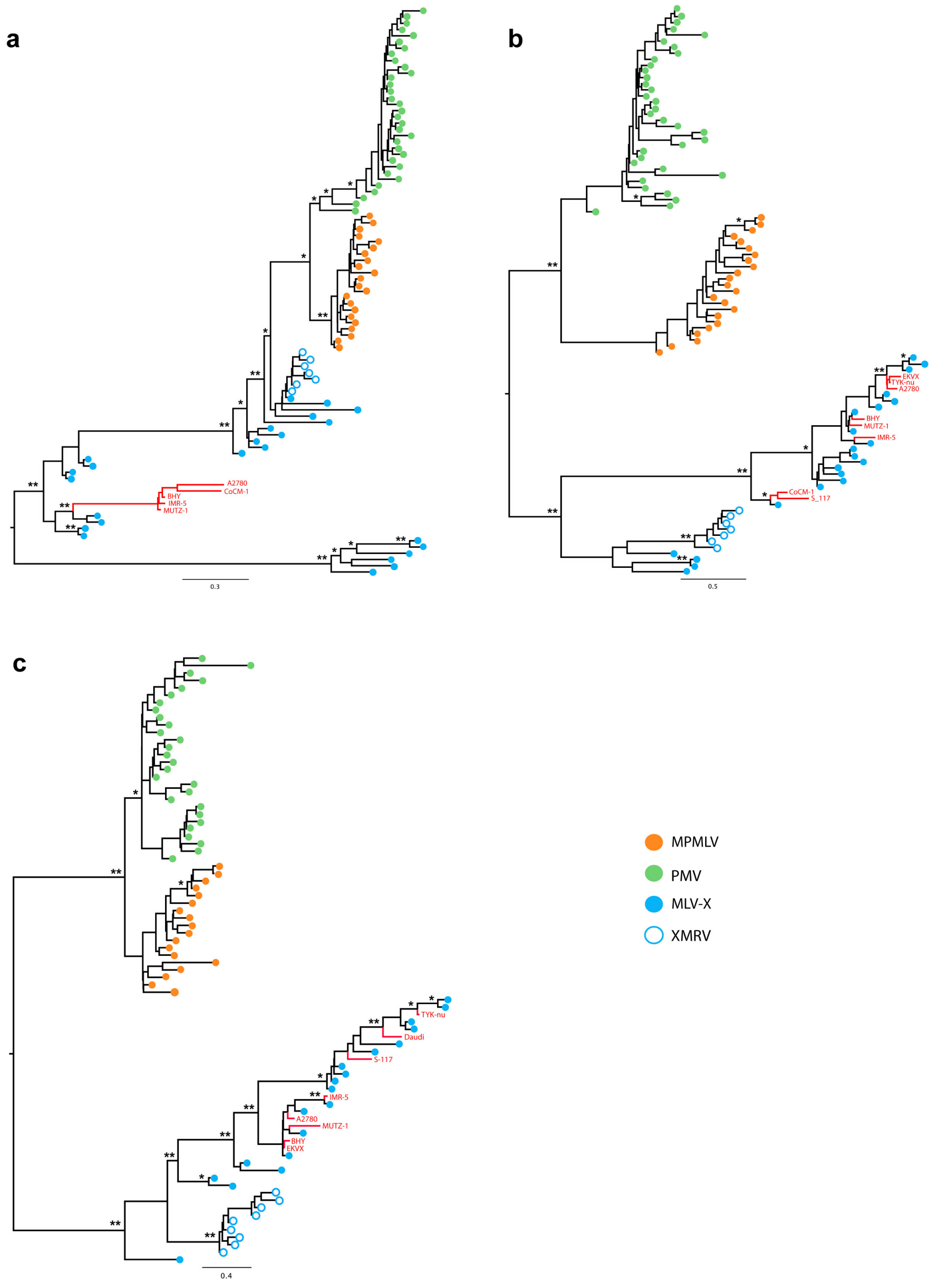

Figure S1. Bayesian maximum clade credibility trees based on the *gag* (a), *pol* (b) and *env* (c) loci of known MLV and MLV-X found contaminating human tumour cell lines. Xenotropic MLV (MLV-X), Polytropic MLV (PMLV) and Modified polytropic MLV (MPMLV) are shown in blue, green and orange circles respectively. XMRVs are represented by blue open circles. MLV-X in the cancer cell lines are indicated in red, the corresponding branch labelled with the cell line name. Bayesian posterior probabilities > 0.90 or 1.00 are indicated on the branches by one or two stars respectively. The scale bar represents the number of nucleotide substitutions per site.
