# Supplementary material for: Disease-associated XMRV sequences are consistent with laboratory contamination
Source: Retrovirology. 2010 Dec 20;7:111. doi: 10.1186/1742-4690-7-111 (PMC3018392; doi:10.1186/1742-4690-7-111)
Supplement: Additional file 5 — Figure S3: Bayesian maximum clade credibility phylogeny of 22Rv1 cell line derived XMRV clones, patient derived XMRV sequences and other murine leukaemia viruses based on the (a) gag, (b) pol and (c) env genetic regions only. Xenotropic MLV (MLV-X), polytropic MLV (PMLV), and modified polytropic MLV (MPMLV) were added as controls. Sequences derived from prostate cancer patients (VP and WO) and chronic fatigue syndrome patients (WPI) are indicated by red and yellow circles respectively. Gene sequences derived from 22Rv1 clones are indicated by blue squares. The trees are rooted by the mid-point rooting method. Bayesian posterior probabilities > 0.95 (*) and of 1.00 (**) are indicated on the corresponding branches. The branching order of the sequences within the XMRV clusters is not statistically supported and therefore cannot be determined unambiguously from these trees. For this reason we have reconstructed a Bayesian phylogeny from the fragments together with the full-length XMRV sequences (Figure 2). The scale bar represents the number of nucleotide substitutions per site. [file 1742-4690-7-111-S5.PDF]

Fig.S3a (*gag*)

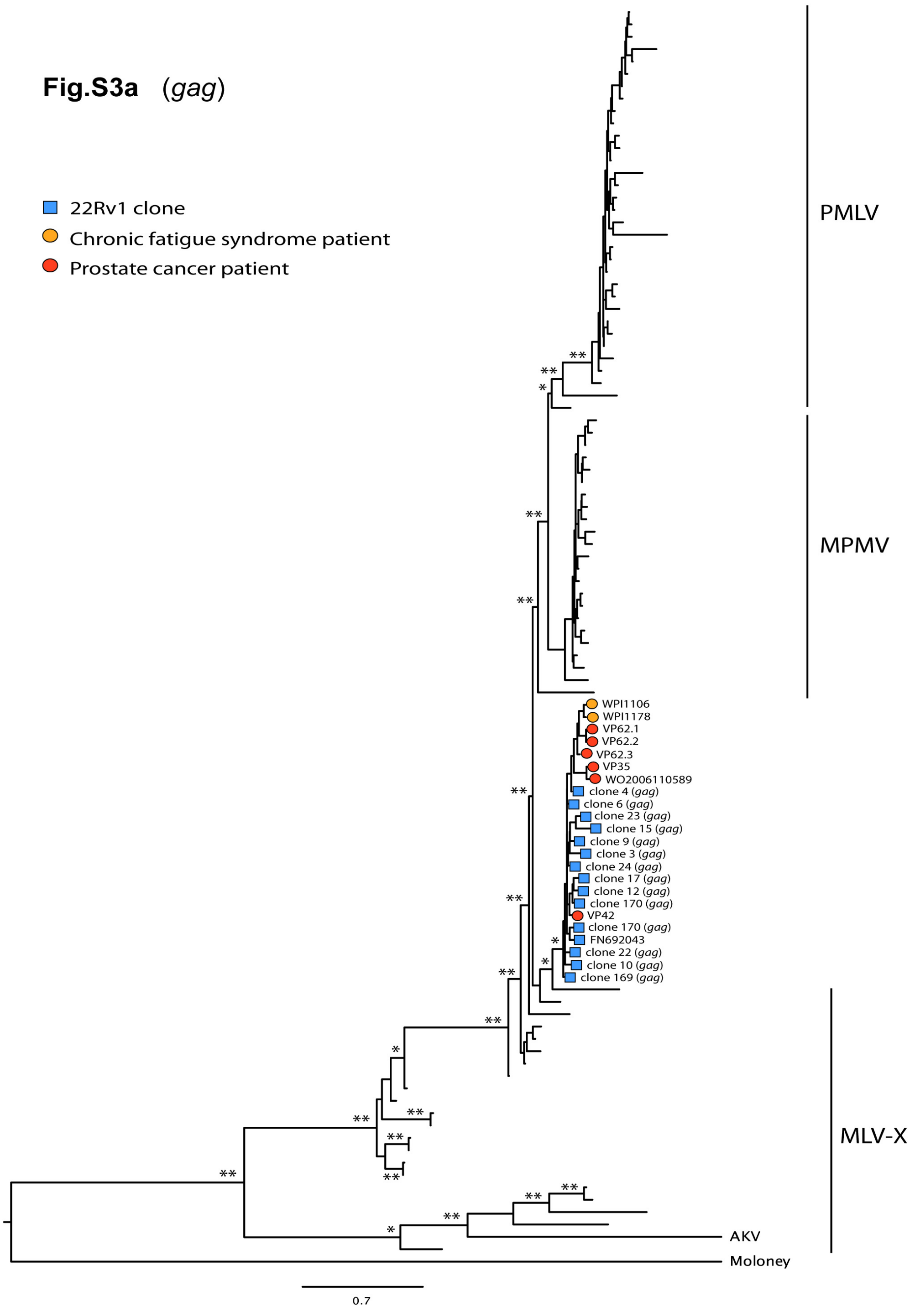
22Rv1 clone
Chronic fatigue syndrome patient
Prostate cancer patient
PMLV
MPMV
MLV-X
WPI1106
WPI1178
VP62.1
VP62.2
VP62.3
VP35
WO2006110589
clone 4 (gag)
clone 6 (gag)
clone 23 (gag)
clone 15 (gag)
clone 9 (gag)
clone 3 (gag)
clone 24 (gag)
clone 17 (gag)
clone 12 (gag)
clone 170 (gag)
VP42
clone 170 (gag)
FN692043
clone 22 (gag)
clone 10 (gag)
clone 169 (gag)
AKV
Moloney
0.7

Fig.S3b (*pol*)

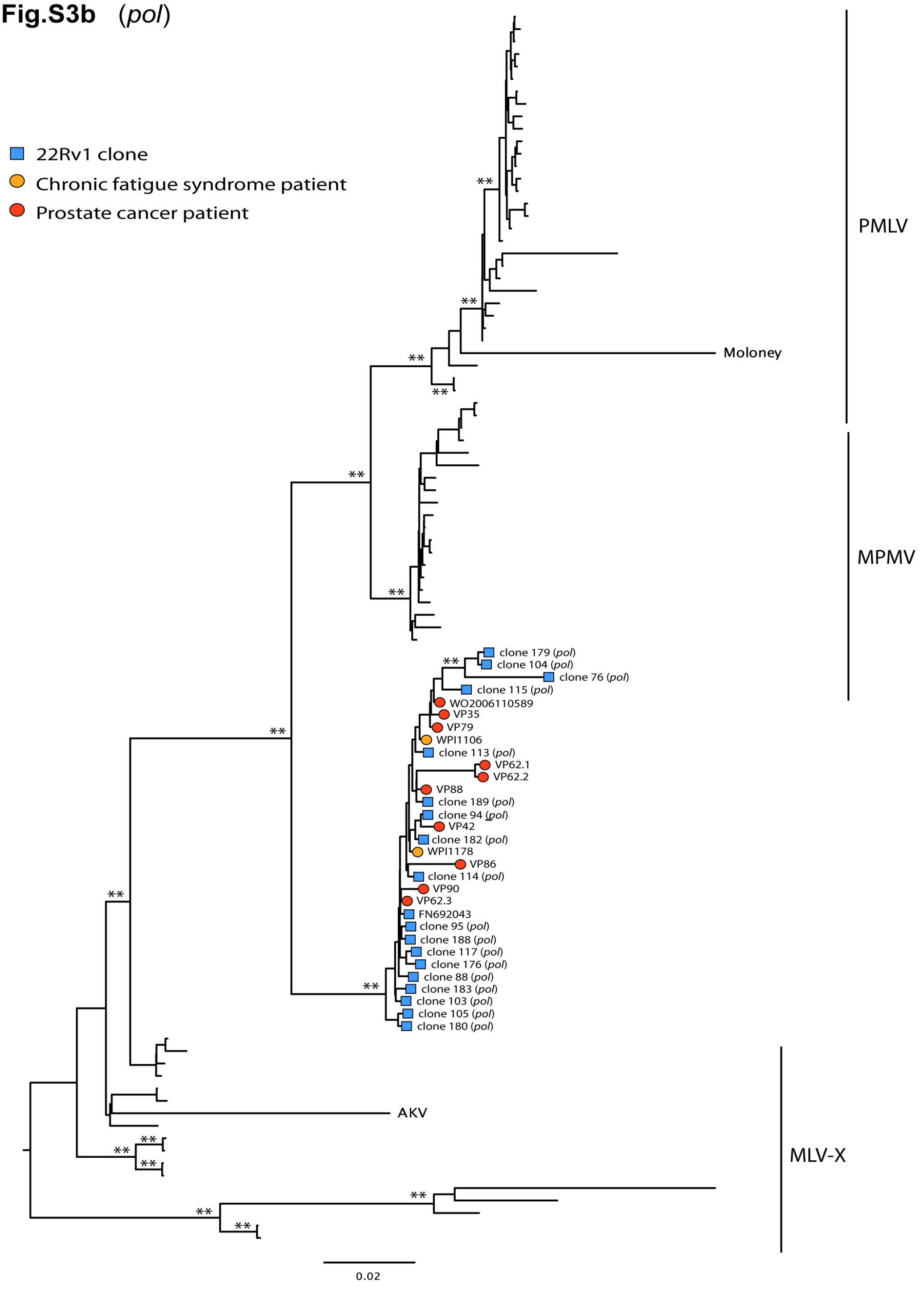

22Rv1 clone
Chronic fatigue syndrome patient
Prostate cancer patient
PMLV
MPMV
MLV-X
Moloney
AKV
clone 179 (pol)
clone 104 (pol)
clone 76 (pol)
clone 115 (pol)
WO2006110589
VP35
VP79
WPI1106
clone 113 (pol)
VP62.1
VP62.2
VP88
clone 189 (pol)
clone 94 (pol)
VP42
clone 182 (pol)
WPI1178
VP86
clone 114 (pol)
VP90
VP62.3
FN692043
clone 95 (pol)
clone 188 (pol)
clone 117 (pol)
clone 176 (pol)
clone 88 (pol)
clone 183 (pol)
clone 103 (pol)
clone 105 (pol)
clone 180 (pol)
0.02

Fig.S3c (*env*)

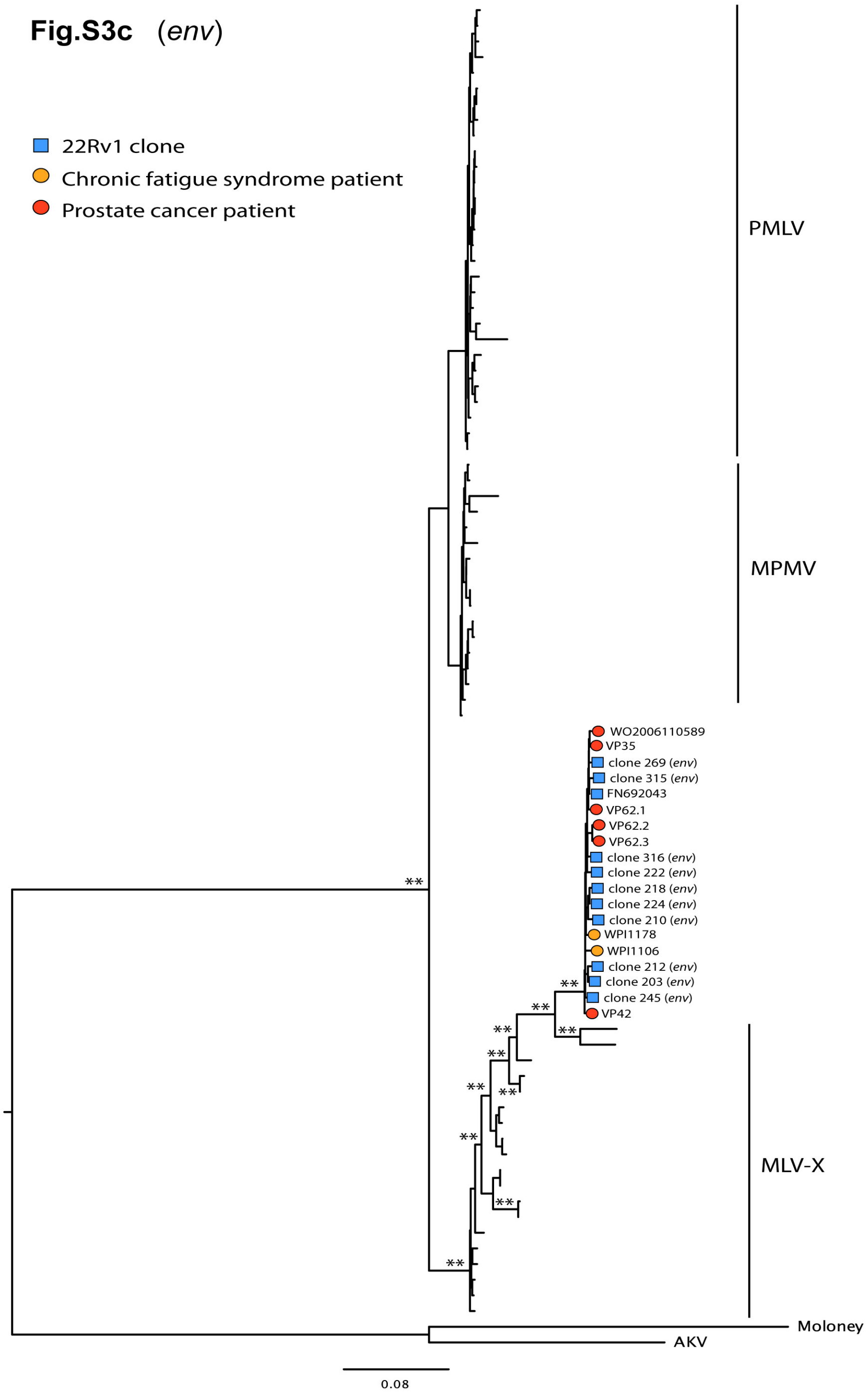

Figure S3. Bayesian maximum clade credibility phylogeny of 22Rv1 cell line derived XMRV clones, patient derived XMRV sequences and other murine leukaemia viruses based on the (a) ***gag*****, (b)** ***pol*** **and (c)** ***env*** **genetic regions only.** Xenotropic MLV (MLV-X), polytropic MLV (PMLV), and modified polytropic MLV (MPMLV) were added as controls. Sequences derived from prostate cancer patients (VP and WO) and chronic fatigue syndrome patients (WPI) are indicated by red and yellow circles respectively. Gene sequences derived from 22Rv1 clones are indicated by blue squares. The trees are rooted by the mid-point

rooting method. Bayesian posterior probabilities > 0.95 (*) and of 1.00 (**) are indicated on the corresponding branches. The branching order of the sequences within the XMRV clusters is not statistically supported and therefore cannot be determined unambiguously from these trees. For this reason we have reconstructed a Bayesian phylogeny from the fragments together with the full-length XMRV sequences (Fig. 2). The scale bar represents the number of nucleotide substitutions per site.
