# Supplementary material for: Disease-associated XMRV sequences are consistent with laboratory contamination
Source: Retrovirology. 2010 Dec 20;7:111. doi: 10.1186/1742-4690-7-111 (PMC3018392; doi:10.1186/1742-4690-7-111)
Supplement: Additional file 6 — Table S3: Genetic diversity of the cell-line and patient-derived gag, pol and env gene sequences. Genetic distances were calculated as i) the observed number of nucleotide substitutions per sites and ii) under the General Time Reversible model of nucleotide substitutions. The significance of difference in the mean genetic diversity between cell line- and patient-derived sequences was tested by Wilcoxon sum rank test. [file 1742-4690-7-111-S6.DOC]

**Table S3. Genetic diversity of the cell-line and patient-derived *gag*, *pol* and *env* gene sequences**

| locus | origin | n | length (nt) |  |  | raw^1^ |  |  |  |  |  | GTR^3^ |  |  |  |
| --- | --- | --- | --- | --- | --- | --- | --- | --- | --- | --- | --- | --- | --- | --- | --- |
|  |  |  |  | Min | 1st Qu. | Mean | 3rd Qu. | Max | p value^2^ | Min | 1st Qu. | Mean | 3rd Qu. | Max | p value^2^ |
|  |  |  |  |  |  |  |  |  |  |  |  |  |  |  |  |
| *gag* | 22Rv1 clones | 16 | 1605 | 0.000 | 0.001 | 0.004 | 0.002 | 0.020 | 0.076 | 0.000 | 0.001 | 0.004 | 0.002 | 0.020 | 0.078 |
|  | Patients | 8 | 1692 | 0.000 | 0.000 | 0.001 | 0.002 | 0.003 |  | 0.000 | 0.0000 | 0.001 | 0.002 | 0.003 |  |
|  |  |  |  |  |  |  |  |  |  |  |  |  |  |  |  |
| *pol* | 22Rv1 clones | 18 | 1635 | 0.000 | 0.002 | 0.003 | 0.003 | 0.008 | 0.005 | 0.000 | 0.002 | 0.003 | 0.003 | 0.008 | 0.027 |
|  | Patients | 13 | 1635 | 0.000 | 0.000 | 0.002 | 0.003 | 0.007 |  | 0.000 | 0.000 | 0.002 | 0.003 | 0.007 |  |
|  |  |  |  |  |  |  |  |  |  |  |  |  |  |  |  |
| *env* | 22Rv1 clones | 10 | 1935 | 0.000 | 0.002 | 0.002 | 0.003 | 0.004 | <0.001 | 0.000 | 0.002 | 0.003 | 0.003 | 0.004 | <0.001 |
|  | Patients | 8 | 1935 | 0.000 | 0.001 | 0.001 | 0.002 | 0.002 |  | 0.000 | 0.001 | 0.001 | 0.002 | 0.002 |  |
|  | | | | | | | | | | | | | | | |
| ^1^ Observed number of nucleotide substitutions per site | | | | | | | | | | | | | | | |
| ^2^ Wilcoxon sum rank test | | | | | | | | | | | | | | | |
| ^3^ Distances corrected according to the General Time Reversible model of nucleotide substitutions | | | | | | | | | | | | | | | |

Genetic distances were calculated as i) the observed number of nucleotide substitutions per sites and ii) under the General Time Reversible model of nucleotide substitutions. The significance of difference in the mean genetic diversity between cell line- and patient-derived sequences was tested by Wilcoxon sum rank test.
