# Supplementary material for: Disease-associated XMRV sequences are consistent with laboratory contamination
Source: Retrovirology. 2010 Dec 20;7:111. doi: 10.1186/1742-4690-7-111 (PMC3018392; doi:10.1186/1742-4690-7-111)
Supplement: Additional file 4 — Figure S2: Maximum likelihood trees showing recombination between XMRV and MoMLV in the sequences derived from PC patients VP29 and VP184. Between nucleotide positions 2400 and 3585 (GenBank Acc. No. AF033811) of the MoMLV pol gene, VP29 and VP184 pol genes are closely related to MoMLV (bootstrap support: 100%) (A), while between positions 3586 to 4921, the same patients derived sequences fall within the XMRV cluster (bootstrap score: 100%) (B). Bootstrap scores above 50% are indicated on the corresponding branches. The scale bar represents the number of nucleotide substitutions per site. [file 1742-4690-7-111-S4.PDF]

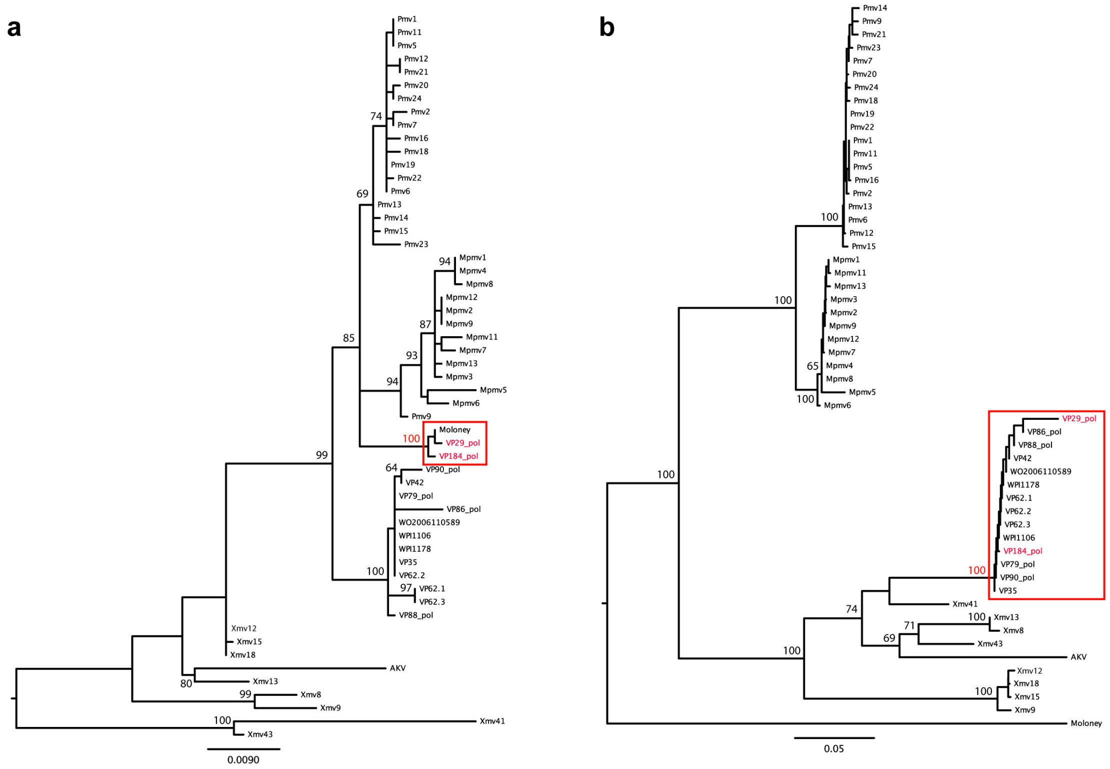

Figure S2. Maximum likelihood trees showing recombination between XMRV and MoMLV in the sequences derived from PC patients VP29 and VP184. Between nucleotide positions 2400 and 3585 (Genbank Acc. No. AF033811) of the MoMLV *pol* gene, VP29 and VP184 pol genes are closely related to MoMLV (bootstrap support: 100%) (A), while between positions 3586 to 4921, the same patients derived sequences fall within the XMRV cluster (bootstrap score: 100%) (B). Bootstrap scores above 50% are indicated on the corresponding branches. The scale bar represents the number of nucleotide substitutions per site.
